# Development of an intervention for patients following an anterior cruciate ligament rupture: an online nominal group technique consensus study

**DOI:** 10.1136/bmjopen-2023-082387

**Published:** 2024-07-18

**Authors:** Hayley Carter, David Beard, Paul Leighton, Fiona Moffatt, Benjamin E Smith, Kate E Webster, Phillipa Logan

**Affiliations:** 1 Physiotherapy Outpatients, University Hospitals of Derby and Burton NHS Foundation Trust, Derby, UK; 2 School of Medicine, University of Nottingham, Nottingham, UK; 3 Surgical Intervention Trials Unit, Botnar Research Centre, NDORMS, University of Oxford, Oxford, UK; 4 School of Health Sciences, University of Nottingham, Nottingham, UK; 5 School of Allied Health, Human Services and Sport, La Trobe University, Melbourne, Victoria, Australia

**Keywords:** Musculoskeletal disorders, Knee, Adult orthopaedics, Orthopaedic sports trauma

## Abstract

**Objectives:**

(1) To develop an intervention for to support patients diagnosed with an anterior cruciate ligament (ACL) rupture with decision-making regarding treatment. (2) To define evidence-based recommendations for the treatment of patients following an ACL rupture.

**Design:**

Nominal group technique consensus study.

**Setting:**

Online meetings with patients and key stakeholders working and receiving treatment in the National Health Service, UK.

**Participants:**

Consensus meetings composed of eight voting participants and five non-voting facilitators. Voting participants included five clinicians, one outpatient therapy manager and two patients with experience in an ACL rupture and reconstructive surgery. Non-voting facilitators supported group discussions and/or observed study procedures. This included a clinical academic expert, two methodology experts and two patient representatives.

**Method:**

Two online meetings were held. Pre-elicitation material was distributed ahead of the first meeting. Premeeting voting was conducted ahead of both meetings. A draft of the shared decision-making intervention and recommendations were shared ahead of the second meeting. Components were discussed and ranked for inclusion based on a 70% agreement threshold.

**Results:**

The meetings led to the development of a shared decision-making intervention to support treatment decision-making following an ACL rupture. The intervention includes two components: (1) a patient information leaflet and key questions diagram and (2) option grid. The evidence-based recommendations encompass core components of treatment reaching the 70% threshold agreed by the group. The recommendations cover: (1) advice and education, (2) exercise guidance, (3) intervention delivery, (4) outcome measure use and (5) shared decision-making.

**Conclusion:**

This study has successfully developed a shared decision-making intervention to support ACL treatment decision-making, ready for testing in a future feasibility study. Evidence-based recommendations for the treatment of patients following ACL injury, ready for testing in a National Health Service (UK) setting, are also presented.

**Trial registration number:**

NCT05529511.

**STRENGTHS AND LIMITATIONS OF THIS STUDY**

- Up-to-date evidence and expert stakeholder opinion (from diverse geographical locations within the UK) were combined to develop the treatment recommendations and shared decision-making intervention.
- The study was underpinned by the extended normalisation process theory to ensure implementation factors were considered and embedded within the development process.
- The recommendations are ready for adoption in clinical practice in the National Health Service in the UK. While developed for this setting, they may be appropriate for use elsewhere with some adaptation.
- The acceptability and effectiveness of the shared decision-making intervention are unknown, future feasibility work is being planned.
- Meetings were held online which may have impacted group dynamics, differing the results from that which may have been produced face to face. Further, there were more healthcare professionals than patient participants which may have altered the power dynamic of the group.

## Introduction

 Anterior cruciate ligament (ACL) tears are common knee injuries affecting more than 20 000 people in the UK each year.^1^ Injury management can follow a surgical (ACL reconstruction, ACLR), non-surgical (rehabilitation) or combined (rehabilitation followed by surgical intervention) pathway. A recent randomised controlled trial (RCT) (ACL SNNAP, 2022) compared surgery with rehabilitation in a UK National Health Service (NHS) setting.^2^ ACL SNNAP reported improved outcomes at 18 months in favour of surgery for self-reported knee outcomes, quality of life and activity level. Further, a 2021 RCT in the Netherlands concluded that early surgery may be better than delayed surgery (although there is limited transferability of this finding to an NHS setting where expediting surgery is not currently feasible due to the backlog in elective orthopaedic procedures following COVID-19).^3^ However, a 2010 RCT in Sweden concluded that surgery may not be appropriate for everyone and engaging in rehabilitation as first-line treatment may avoid unnecessary surgical intervention.^4^ Understanding which patients are appropriate for surgical or non-surgical treatment is challenging. There is uncertainty over treatment decision-making and the sequence of specific treatments and any related temporal factors (ie, when to undergo specific treatments) continue to be debated.

Shared decision-making (SDM) tools are a decision aid that may help patients make decisions about their health and treatment. SDM is outlined as a key element of healthcare by both the National Institute of Care and Excellence and NHS England board.^5 6^ It is defined as a collaborative process between patients and clinicians to determine investigations, management plans and support needed based on individual preferences and relevant evidence.^7^ SDM regarding treatment for ACL tears is especially important as there is uncertainty reported in the literature about treatment efficacy.

In addition to uncertainty about treatment decision-making, optimum treatment for patients following an ACL rupture is unknown. Although best practice guidance was jointly published by the British Orthopaedic Association, British Association for Surgery of the Knee and British Orthopaedic Sports Trauma Arthroscopy Association in 2020, detailed recommendations regarding the delivery of advice and education, rehabilitation, mediums of treatment and outcome measure use are not included.^8^ Therefore, clinical practice in the UK varies and optimum treatment is unknown.^9^

The purpose of this study was to develop a theoretically informed SDM intervention, using an online video chat nominal group technique (NGT), to support patients with decision-making regarding treatment post-ACL rupture. We also aimed to define treatment recommendations for patients proceeding with rehabilitation and/or surgical intervention to support standardisation of treatment across the UK. The developed SDM intervention will later be tested in a feasibility trial. The extended normalisation process theory (ENPT) was used as the underpinning theory for the study.^10^ The use of theory to support intervention development is recommended by the Medical Research Council and National Institute for Health Research framework for developing and evaluating complex interventions, however, no single theory has been identified as superior to another.^11^ A recent review exploring the normalisation process theory/ENPT’s use in interventional research of musculoskeletal/orthopaedic conditions found it to be compatible for use across a variety of conditions and healthcare settings and encouraged its use during intervention development processes.^12^

## Method

### Study design

This study used the NGT method to:

1. Develop an SDM intervention for use with patients following diagnosis of an ACL rupture.
2. Gain consensus for treatment recommendations covering five domains: advice and education, exercise guidance, delivery method, outcome measure use and SDM.

**Figure 1:**
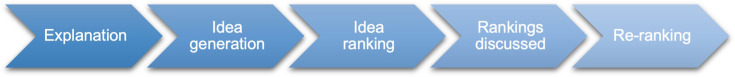
Nominal group technique process.

NGT uses group meetings comprising appropriate experts to gain consensus on a topic area.^13^ This approach has commonly been used in healthcare intervention development.^13 14^ Although usually face to face, the NGT method was adapted to be conducted online via video teleconference to support collaboration from participants in a spread of geographical locations. This has previously been identified as a pragmatic concern of the face-to-face method.^15^ Successful online NGT studies have also been described elsewhere.^16^ The online NGT process followed the same structure as described for face to face, this is shown in figure 1.

### Sample size and recruitment

Since the development of the NGT method in 1971, there has been debate about the optimal sample size for meetings.^17^ More recent literature has proposed 6–12 participants with suggestions that below 6 will reduce reliability and above 12 will diminish the improvements in reliability.^15 18^ We, therefore, aimed to recruit a sample of 6–12 participants, with a minimum of one participant from each identified professional or patient group. Participants eligible for inclusion included NHS healthcare professionals (HCPs) with a special interest/expertise in treating ACL injuries, therapy managers of an NHS musculoskeletal outpatient department and patients who were awaiting or who had undergone an ACLR.

Participants volunteered to take part in response to study material advertised via professional and special interest networks (Association for Trauma and Orthopaedic Chartered Physiotherapists and through interested clinicians), social media (Twitter: @POP_ACLR) and an existing patient and public involvement and engagement (PPIE) group. The sampling strategy was to include 6–12 participants with a minimum of one patient, one HCP and one therapy manager. Where more than one potential participant volunteered, their characteristics were considered to ensure variation in group expertise and experience. For example, we aimed to include HCPs from different backgrounds such as medical and allied health professionals.

The consensus group comprised voting and non-voting participants. Overall, there were 13 participants. The voting participants included one orthopaedic consultant, three orthopaedic/musculoskeletal physiotherapists, one occupational therapist specialised in vocational rehabilitation in a musculoskeletal outpatient therapy department, one outpatient therapy manager who was also a physiotherapist and had lived experience of an ACLR and two patients with lived experience of an ACL rupture and reconstructive treatment. Non-voters did not partake in voting due to engagement in other research related to this study (this paper reports the intervention development phase of a wider mixed-methods study). The non-voting participants included one clinical academic with expertise in orthopaedic and musculoskeletal research including ACL injuries and treatment; two of the lead researcher’s PhD supervisors (one with experience in conducting NGT studies and one who is also a clinical academic musculoskeletal physiotherapist) and two patient representatives who have been involved in the wider study and its setup. The clinical academic expert was included to support communication of the most up-to-date literature. The lead researcher’s PhD supervisor with experience in NGT methods was present to observe the group, providing oversite to methodological rigour and to support reflexive practices. The patient representatives were involved to support patient participants as they have experience in discussing their views at trial steering committee meetings and PPIE workshops with patients and clinicians.

The meetings were chaired by the lead researcher who was a practising physiotherapist. Three voting participants (surgeon, therapy manager and patient) and one facilitator (PhD supervisor/clinical academic) were unable to make the second meeting due to other commitments. These participants were still included in the premeeting voting ahead of the second meeting.

### Data collection and analysis

Two meetings were held via Microsoft Teams. All participants gave consent to participate in the meetings which were also recorded for study purposes. The first meeting lasted 1 hour 51 min, the second meeting lasted 2 hours.

Prior to the first meeting, participants were provided with relevant, up-to-date literature in addition to unpublished results from a preceding study which explored patients lived experiences of the ACL surgical treatment pathway pre- and post-surgery using qualitative interviews. All participants received the same information, with a lay document provided to non-clinical participants (available in online supplemental file 1 (HCPs) and online supplemental file 2 (lay version)). In addition to the information document, premeeting voting was conducted to support discussions at the first meeting via Microsoft Forms. Participants were asked to vote on a 5-point Likert scale whether components were ‘very important’, ‘important’, ‘neither important nor not important’, ‘not important’ or ‘not at all important’ for inclusion. Participants voted on five components: (1) advice and education, (2) exercise guidance, (3) delivery method, (4) outcome measures and (5) decision-making. A blank copy of the voting form is available in online supplemental file 3. Free-text responses were also permitted to stimulate further thought and discussion points to allow room for additional components and topics to be added. This supported the first two stages of the NGT process: explanation and silent generation of ideas.

The purpose of the first meeting was to gain consensus on core components for the recommendations and SDM intervention. A 70% cut-off was used to include components that were considered ‘very important’. Components were deemed ‘important’ if 70% was reached combining scores of very important and important. Components below 30% were excluded and those not reaching the 70% inclusion cut-off nor the 30% exclusion were discussed. This threshold was agreed by the study team in line with other consensus recommendations.^19 20^ During the meeting, Mentimeter was used for anonymous voting and idea sharing. The first meeting consolidated the five components of the recommendations, with no additional components suggested for inclusion. The ‘very important components’ were considered to be essential while the ‘important’ components were recommended to support the personalisation of care. The recommendations are evidence based and theoretically informed and aim to offer pragmatic guidance to ensure they are achievable, applicable and relevant to the UK NHS setting. The decision-making discussions were split into elements to be included in the recommendations and specific design of the SDM intervention. The need for the recommendations was felt to be important as a direct impact on clinical practice ahead of future feasibility testing of the SDM intervention.

A draft version of the recommendations and SDM intervention was shared with participants ahead of meeting 2 to provide a basis for group discussion. A second round of voting among NGT group participants was conducted prior to the second meeting to support idea sharing and generate discussion. A blank copy is available in online supplemental file 4. The second meeting was conducted to agree on specific elements of each component to finalise the recommendations and SDM intervention. The parameters for inclusion were the same as that used for meeting 1. At the end of the second meeting, the overall components and specific elements for the SDM intervention and recommendations were finalised.

The final version of the SDM intervention and treatment recommendations was communicated with all NGT group participants after meeting 2 (including those who were unable to attend). No concerns were raised by any participant so the final versions were considered representative of the discussions and voting across both meetings.

**Figure 2:**
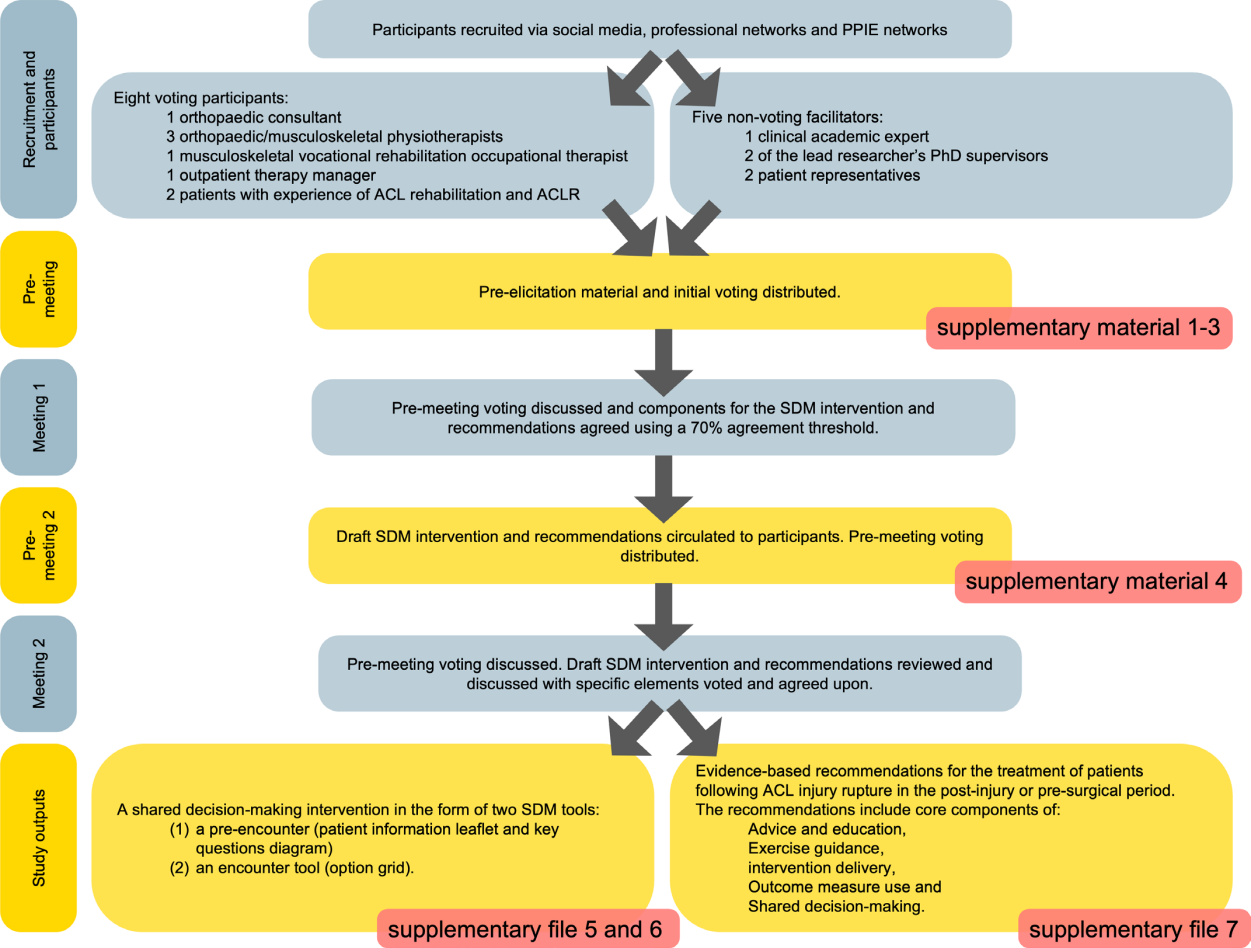
Overview of study process. ACL, anterior cruciate ligament; ACLR, ACL reconstruction; SDM, shared decision-making.

An overview of the study process is shown in figure 2 (including onlinesupplemental files 1^7^).

### Guidelines and supporting theory

The International Patient Decision Aid Standards criteria and option grid development guidelines were used to support the development of the SDM intervention.^21^,^23^ The National Institute for Health and Care Excellence framework for development of decision aids was also used.^24^ Visual representation of how these were used is available in online supplemental file 8. The implementation research logic model (IRLM) was used to support visual representation of the SDM intervention considering future implementation and evaluation in a feasibility study.^25^ ENPT was used to stimulate discussions during NGT meetings about implementation to embed these considerations in the development process. The four constructs of ENPT were used to consider how the intervention would integrate with current NHS systems/pathways (capability), the users’ (ie, clinicians and patients) ability to engage with and deliver the intervention (eg, considering their resources and skillset) (capacity and potential) and the work and ability of the users to continue engaging with the intervention to allow it to become part of normal practice (contribution). The implementation strategies as part of the IRLM were also mapped to ENPT. The mapping of implementation strategies to ENPT and IRLM is available in online supplemental file 9.

### Patient and public involvement

Patients and key stakeholders (including orthopaedic surgeons, therapy managers and therapists) with experience in having, treating or managing departments treating ACL injuries were involved in the grant funding application, study design and setup. This supported research decision-making regarding participant eligibility criteria, sample size for the study, the research methods and analytical approach.

## Results

We present the results for:

1. An SDM intervention comprising two components: (1) a pre-encounter tool (a patient information leaflet and key questions diagram) and (2) an encounter tool (option grid).
2. Evidence-based recommendations for the treatment of patients following ACL rupture in the postinjury or presurgical period. These recommendations cover core components of advice and education, exercise guidance, intervention delivery, outcome measure use and SDM.

### SDM intervention

The SDM intervention is available in onlinesupplemental files 4  5.

The NGT meeting focused on designing an intervention to support SDM regarding treatment following an ACL rupture. The SDM intervention was split into two components: (1) pre-encounter and (2) encounter. Two pre-encounter tools were developed, designed for use by patients prior to a clinical consultation. The pre-encounter tool comprises a patient information leaflet and a diagram to support patients to consider key questions about their treatment. The key questions diagram will be included in the patient information leaflet to form one tool. The encounter tool, an option grid, was designed for use during clinical consultations by patients and HCPs. The tools were developed using the agreed SDM recommendations by the group in meeting 1 (presented below). Prior to meeting 2, participants were sent drafts of the three tools for comments, with suggested amendments discussed and voted on during the second meeting. The tools were also mapped to the IRLM to support consideration of the key determinants, implementation strategies, mechanisms and outputs.^25^ A copy of the IRLM for the study is included in online supplemental file 9. This was presented to participants at meeting 2. Key areas that were refined include adding: (1) detail to the information leaflet about the physical and mental effort of ACL injury treatment (online supplemental file 5, page 3) (2) information about the increased risk of osteoarthritis following ACL injury to the information leaflet (online supplemental file 5, page 6), (3) questions about the potential loss of income, employer sickness policy and how treatment may impact an individual’s life to the key questions diagram (online supplemental file 5, page 8), (4) information about fitness management to the option grid (online supplemental file 6, page 2) and (5) a blank box for the ‘clinician recommendation’ section of the option grid (online supplemental file 6, page 2). The SDM intervention (component 1. Patient information leaflet, including the key questions diagram, component 2. Option grid) will be tested in a future feasibility study in an NHS setting.

### Treatment recommendations

The treatment recommendations are available in online supplemental file 7.

### Advice and education

**Figure 3:**
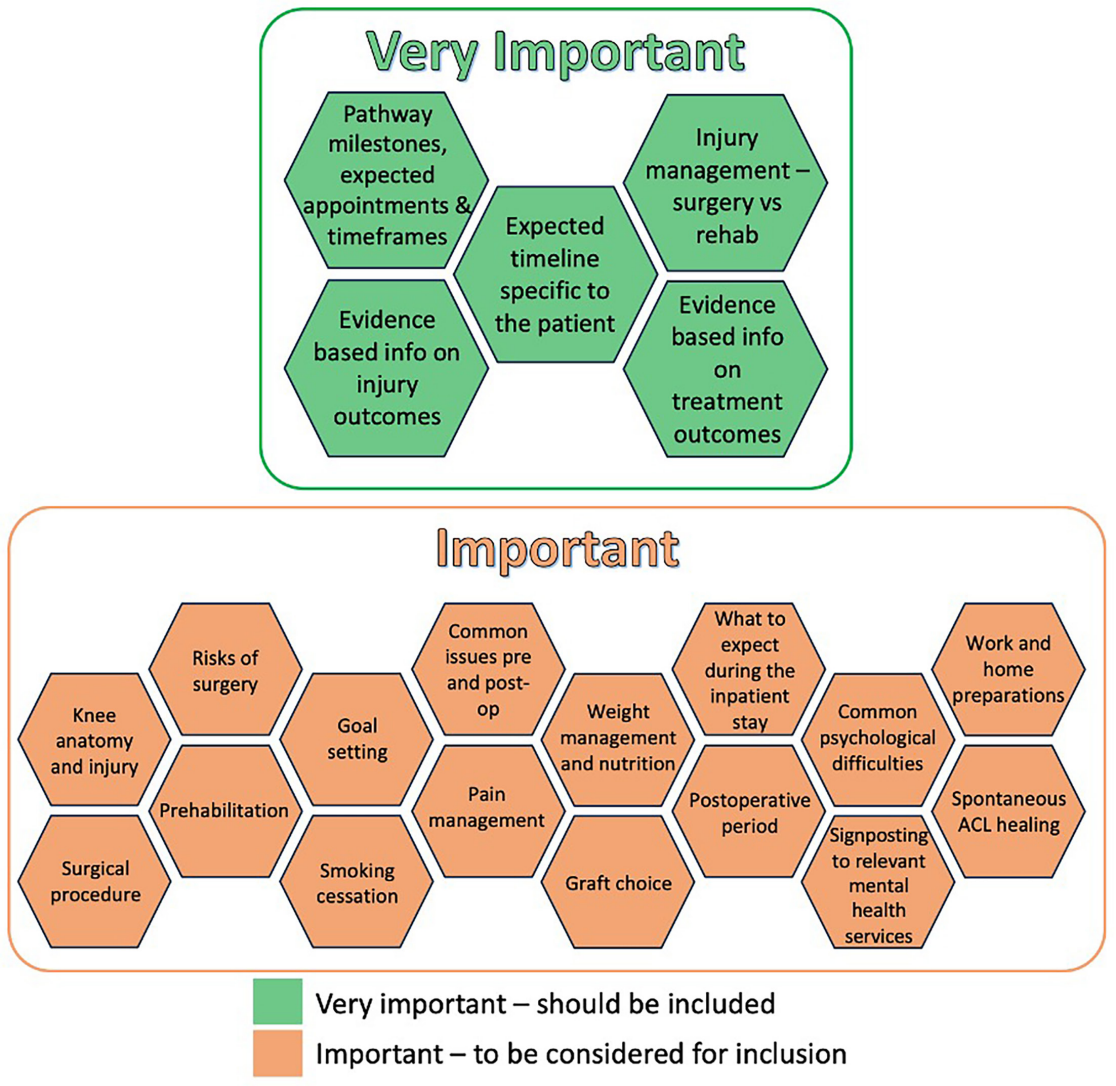
Advice and education topics. ACL, anterior cruciate ligament.

There were 20 advice and education topics voted on ahead of and discussed during the meeting. Five reached the 70% threshold to be considered as ‘very important’ for inclusion and the remaining 15 reached the threshold to be considered ‘important’. A free-text comment suggested the inclusion of spontaneous ACL healing. Following discussion among the group and Mentimeter voting, this was considered to be ‘important’ and so 16 topics were considered to be ‘important’. The final recommendations are shown in figure 3.

The group further discussed the delivery of advice and education, considering it ‘very important’ that it is delivered: (1) in a one-to-one session, (2) by any clinician with expertise and knowledge to do so and (3) consistently by the healthcare team across the patients care.

### Exercise guidance

**Figure 4:**
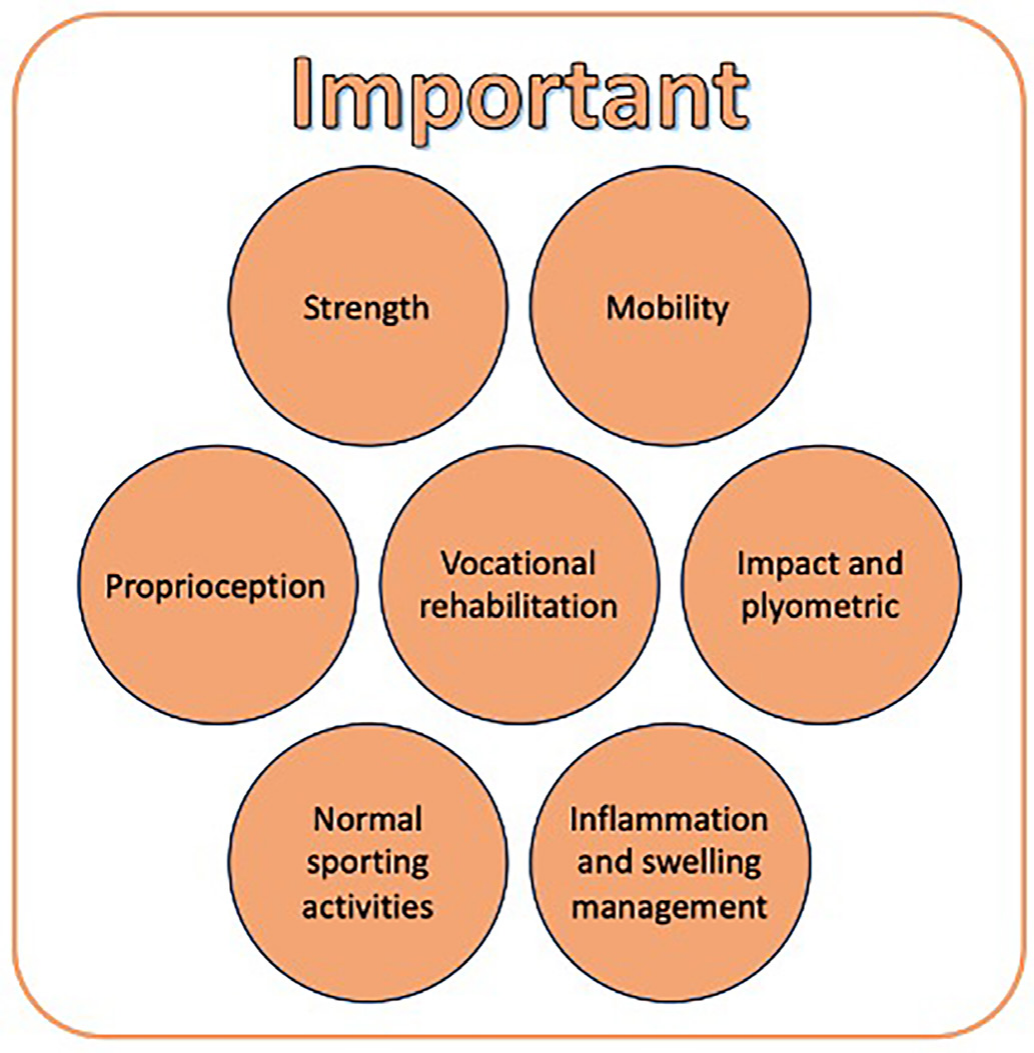
Exercise types to be considered by clinicians.

There were nine exercise topics voted on ahead of and discussed during the first meeting. These included that exercise guidance in the preoperative period should include at least one session with a rehabilitation/exercise therapist, 2–6 sessions with a rehabilitation/exercise therapist, more than six sessions with a rehabilitation/exercise therapist, exercise type(s), frequency of exercise completion, exercise programme length, time period to be completed, delivery method and guidance on exercise/types of activity to avoid. One reached the 70% threshold considered as ‘very important’ for inclusion, that as a minimum, at least one session should be offered with a rehabilitation/exercise therapist within 3 months of ACL injury diagnosis. It was considered ‘important’ that clinicians offer further sessions based on individual patient need, considering specific goals and waiting times for surgery (if appropriate). Specific exercise components were discussed including exercise type, exercise programme frequency and length, and do’s and don’ts. It was agreed specific exercise types should not be recommended to be completed by every patient following ACL injury. Instead, the group offer suggestions of exercise types for considerations by clinicians as appropriate for individual patients and their specific goals. Exercise-type suggestions are shown in figure 4.

**Figure 5:**
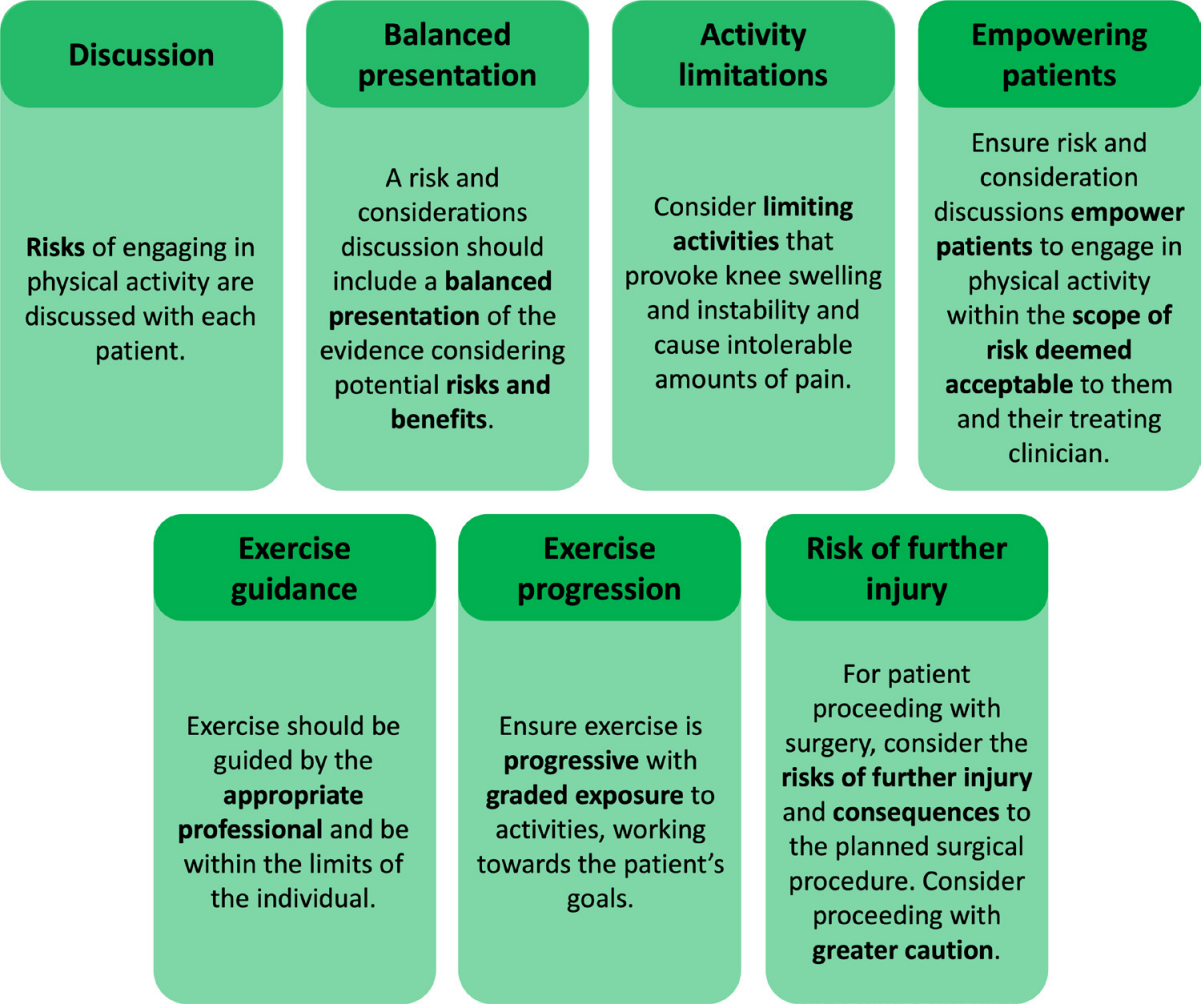
Risks and considerations.

It was considered very important that exercise programmes are completed a minimum of 2–3 times a week and continuously for the preoperative period or until a non-surgically managed patient meets their aims/goals. The group considered ‘do’s and don’ts’ at the second meeting following a number of suggestions at meeting 1. Several considerations and scenarios were discussed. It was agreed that exercise should be guided by the appropriate professional and be within the limits of the individual. While it is acknowledged that activities that provoke knee swelling and instability and cause intolerable amounts of pain may need to be limited, we recommend progressive and graded exposure to activities, working towards the patients’ individual goals. It was further considered very important that the risks of engaging in physical activity are discussed with each patient. This discussion should be based on the patients’ goals to allow them to make educated decisions about engaging in physical activity after their ACL injury. The group discussed the importance of this conversation empowering patients to engage in physical activity within the scope of risk deemed acceptable to them and their treating clinician(s). The discussion should be a balanced presentation of the evidence considering potential risks and benefits of engaging in different activities/exercise types. For patients proceeding with surgery, the group recommend considering the risk of further injury and subsequent consequences for the planned surgical procedure. Expression of caution to return to cutting/pivoting activities may, therefore, be greater with this patient group. The group subsequently agreed to rename these recommendations to ‘risks and considerations’ as opposed to ‘do’s and don’ts’ to reflect the nature of these conversations more accurately. These recommendations are summarised in figure 5.

### Delivery method

The delivery method of interventions post-ACL injury/pre-ACLR was discussed. It was considered important that the following six mediums are considered by clinicians: (1) booklet, (2) website, (3) face to face, (4) one to one (via virtual methods or in-person), (5) peer support and (6) combination of face to face and digital or printed resources. The importance of the delivery method meeting the needs of the individual was discussed and considered a priority for choosing the appropriate method. It was recommended that an initial one-to-one session is offered to ensure specific needs are met and considered when selecting an appropriate medium for intervention delivery.

### Outcome measures

The fourth element presents recommendations for the use of outcome measures for patients following ACL injury. Three measures were considered to be very important, and the group recommend these are used, as a minimum, with all patient’s postinjury regardless of the treatment decision of rehabilitation or surgery. These include: (1) knee-specific outcome measures, (2) psychological outcome measures and (3) current level of activity including occupation. Six further outcome measures were considered to be important and are advised to be used as appropriate for individual patients. Outcome measures to be considered for use include: (1) patient estimated ability to return to their preinjury level of activity, (2) patient expectations of treatment and outcomes, (3) preinjury level of activity, (4) patient satisfaction, (5) weight screening and (6) clinical assessment. The group agreed on the recommendation of these outcome measure groups, rather than selecting a specific measure for each category. This was decided to ensure outcome measure selection is most appropriate for individual patients. It further aligns with the ACL literature where several different outcome measures are recommended for use in this population.^26^ The group agreed that outcome measures should not differ based on the expected treatment of surgery or rehabilitation. Clinicians should consider assessing all patients at baseline to help with future treatment planning and progress tracking.

### Shared decision-making

**Figure 6:**
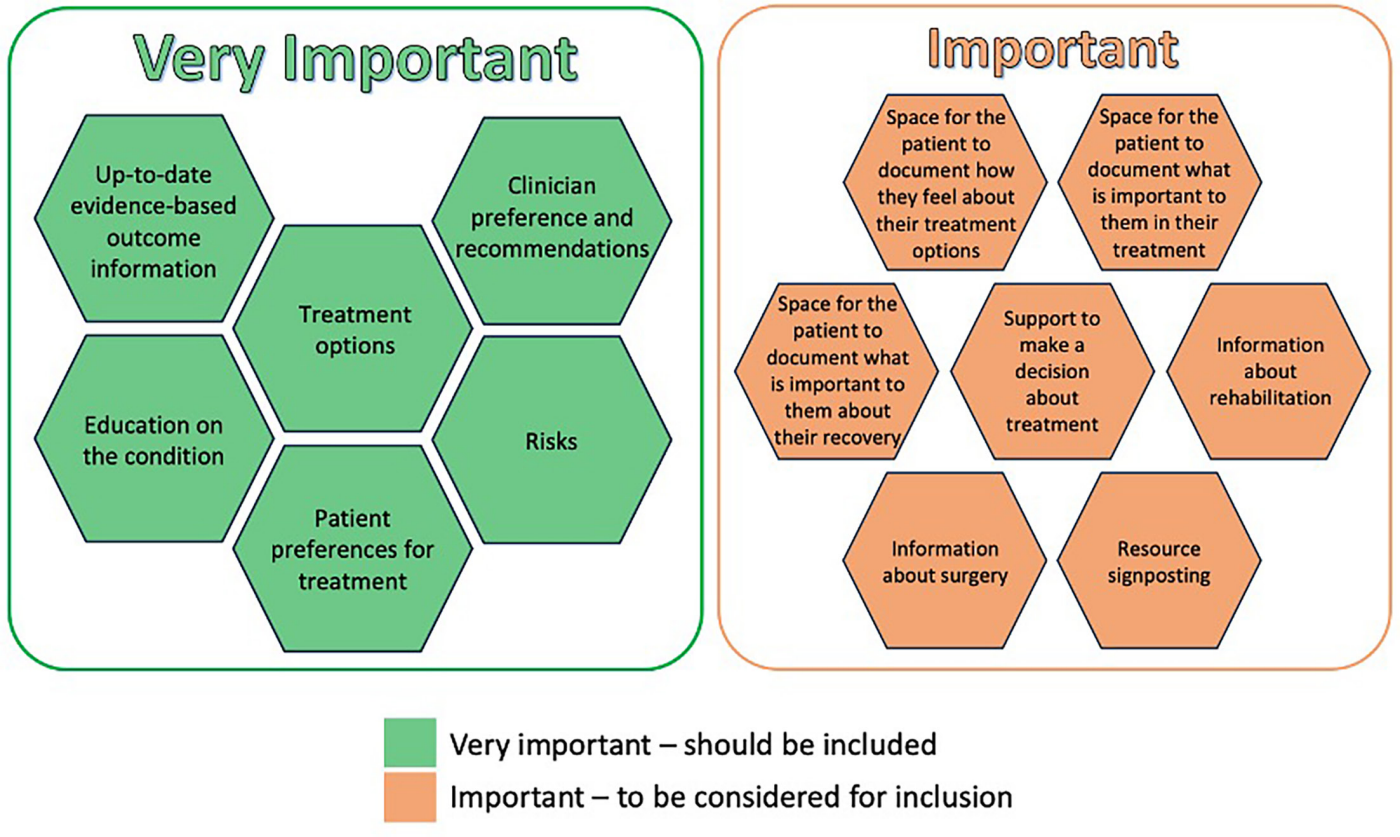
Shared decision-making recommendations.

The final recommendations are regarding SDM. The group discussed the principles of SDM with the aim of determining important components to be included in an SDM tool and/or during a clinical consultation. Six principles were considered to be very important and seven were considered important. These are presented in figure 6.

While a specific SDM intervention has been developed, it was agreed among the group to be beneficial to consider recommendations for SDM principles directly applicable to clinical practice. The need for these recommendations was felt to be important as a direct impact for clinical practice ahead of future feasibility testing of the SDM intervention.

It was considered important that the SDM tool be designed for completion by the HCP and patient together, be available online and via paper and include a flow chart of key questions to help the patient consider their treatment options.

Participants were asked to vote ahead of the second meeting whether the drafted recommendations were representative of discussions at meeting 1. 100% of participants agreed they represented previous discussions. Only minor amendments were made at meeting 2 including: (1) elaboration of the importance of individualised mental health support as part of the advice and education recommendations, (2) consideration of exercise-type selection and (3) addition of risks and considerations in place of ‘do’s and don’ts’. The recommendations have been summarised in a single document including a three-page summary.

## Discussion

This consensus study has led to the development of:

1. An SDM intervention comprising two components: (1) a pre-encounter tool (patient information leaflet and key questions diagram) and (2) an encounter tool (option grid).
2. Evidence-based recommendations for the treatment of patients following ACL injury rupture in the postinjury or presurgical period. These recommendations cover core components of advice and education, exercise guidance, intervention delivery, outcome measure use and SDM.

These outputs were successfully developed using the online NGT approach with key underpinning principles of ENPT. Both outputs were developed for implementation in the NHS, a public healthcare system unique to the UK. The transferability to other healthcare systems may, therefore, be limited. For example, it has previously been reported that patients interact with a median of three different HCPs prior to confirmation of an ACL rupture in the NHS.^27^ MRI waiting times are approximately 6–8 weeks and vary between hospitals, with waiting times for surgery currently upwards of 12 months. While in a private healthcare setting, a patient is likely to access an MRI scan sooner, see fewer HCPs and have the option to proceed with surgical intervention in a shorter timescale.

The SDM tools are a novel development to support injury management decisions for patients following ACL rupture. The patient information leaflet and key questions diagram were designed with the aim of improving patient knowledge; a previously recognised benefit of pre-encounter tools.^28^ As previous research has reported that pre-encounter tools fail to influence SDM practices an encounter tool was also developed in the form of an option grid.^29 30^ A 2015 trial by Elwyn *et al* explored the impact of an option grid for patients diagnosed with knee osteoarthritis in a stepped wedge trial.^31^ Use of the option grid led to significantly increased patient knowledge and readiness to decide on treatment. It was also reported that use of the tool did not lead to an increase in clinical consultation length and was also successfully used with translators for patients whose first language was not English. A potential barrier to SDM practices at present may be a pragmatic concern of time constraints during consultations. During stakeholder engagement and NGT meeting discussions, clinicians reflected positively that previous research has demonstrated no additional burden of using an SDM tool. Two essential elements of SDM include (1) knowledge exchange between parties and (2) patient preferences.^32^,^36^ It is hoped that the use of the pre-encounter tool will support increases in knowledge and the encounter tool will support patients to consider and express their treatment preferences, resulting in informed decision-making and SDM practices during consultations.

The development of these recommendations supports those previously published by Filbay and Grindem in 2019.^37^ Filbay and Grindem’s evidence-based recommendations provide information on injury diagnosis, management, rehabilitation and criteria to inform return to sport. While these previously published recommendations cover a number of areas and summarise current evidence they do not: (1) offer clear topics to be included in advice and education information, (2) offer very important and important elements to guide exercise, (3) consider the delivery method of rehabilitation, (4) recommend outcome measures to be used in clinical practice or (5) recommend very important principles of SDM. These recommendations were also published prior to two RCTs comparing surgery versus rehabilitation for the treatment of ACL injuries and therefore warranted updating.^2 3^ Our study combines this evidence with the voice of key stakeholders ensuring the production of clinically relevant recommendations in the key areas of advice and education, exercise guidance, intervention delivery, outcome measure use and SDM principles. They go further to present essential components for inclusion and components to be considered to allow tailoring for individual care. These recommendations are the first step towards standardising practice in the UK. While the recommendations were developed for use in the UK NHS service, they may be appropriate for use elsewhere in other health settings.

### Strengths, limitations and reflexive considerations

This study included a range of different stakeholders involved in the care of patients with ACL injury and the future implementation of the SDM tools. Patients who may benefit from the SDM tools were also involved as participants and facilitators of the NGT meetings. The involvement of these key stakeholders will support the implementation and acceptability of tools in clinical practice.

As a range of stakeholders were involved in the study, it is important to consider the power dynamic among the group and how this may have influenced the final results. It is acknowledged that there were more HCPs involved in the study than patients. Patients involved in the wider study were invited as facilitators to support the voting patient participants. The patient facilitators have experience in discussing their views at trial steering committee meetings and PPIE workshops with patients and clinicians. Their inclusion hoped to reduce the potential for the patient voice to be lost. As the researcher conducting the meetings was also aware of this, every effort was made to ensure patient participants were active in group discussions. They also had the opportunity to offer anonymous feedback via Mentimeter. An introduction round was also conducted at the start of both meetings to ensure participants were comfortable speaking in front of the group. The use of virtual meetings may have been a barrier for some (patients and HCPs) to participating in discussions but an enabler for others who may have felt less comfortable talking in front of an in-person group. Further, both patients included in the study had experienced ACLR. This may have influenced patient perspectives on important information to be included in the SDM intervention and treatment recommendations, with a potential preference for surgical intervention. The addition of patient participants with experience of non-surgical management may have offered different perspectives and influenced group discussions and study outputs.

A strength of this study was the inclusion of participants in differing geographical locations, ensuring historical practices at one organisation did not dominate discussions and voting. Further, the consensus thresholds set are consistent with established consensus recommendations adding robustness to study procedures.^19 20^ The recommendations produced are based on the current evidence base and expert consensus of patients and HCPs, ready for adoption in clinical practice in the UK. It is important to acknowledge that further evidence published after study completion may alter the recommendations. In addition, feasibility testing of the SDM intervention is needed to determine its acceptability before it can be implemented widely in clinical practice. Funding is in place and ethical approval is being sought for a mixed-methods feasibility study with 20 patients in a UK NHS setting.

ENPT was used as the underpinning theory in this study. A previous systematic review has outlined that ENPT is suited to support the development of musculoskeletal/orthopaedic interventions, however, the report of its use is sparse.^12^ Author reflexivity (an individual’s reflections of their assumptions, feelings, reactions, judgements) of its use has also previously been recognised to be limited.^12 38^ Reflexive practices of the lead researcher were supported by observations of NGT meetings by their PhD supervisor. The lead researcher reflects positively on ENPTs use in enabling greater attention paid to implementation factors during the design process. Embedding the theory into the intervention development process has also allowed for ease of transition towards feasibility planning and is hoped that some implementation barriers have been considered and addressed prior to feasibility testing.

## Conclusion

This study has successfully developed an SDM intervention to support ACL treatment decision-making, ready for testing in a future feasibility study. Evidence-based recommendations for the treatment of patients following ACL injury, ready for testing in a UK NHS setting, have also been defined. These include (1) advice and education, (2) exercise guidance, (3) delivery method, (4) outcome measure use and (5) SDM principles.

## supplementary material

10.1136/bmjopen-2023-082387 online supplemental file 1

10.1136/bmjopen-2023-082387 online supplemental file 2

10.1136/bmjopen-2023-082387 online supplemental file 3

10.1136/bmjopen-2023-082387 online supplemental file 4

10.1136/bmjopen-2023-082387 online supplemental file 5

10.1136/bmjopen-2023-082387 online supplemental file 6

10.1136/bmjopen-2023-082387 online supplemental file 7

10.1136/bmjopen-2023-082387 online supplemental file 8

10.1136/bmjopen-2023-082387 online supplemental file 9

## Data Availability

Data are available on reasonable request.
