# Supplementary material for: Development of an intervention for patients following an anterior cruciate ligament rupture: an online nominal group technique consensus study
Source: BMJ Open. 2024 Jul 18;14(7):e082387. doi: 10.1136/bmjopen-2023-082387 (PMC11261705; doi:10.1136/bmjopen-2023-082387)
Supplement: online supplemental file 1 [file bmjopen-14-7-s001.pdf]

# POP-ACLR Consensus Meeting Pre-Reading
## Information, Decision Making and Treatment

## Table of Contents

*Research aims* ... *1*

*Meeting aims* ... *1*

*Purpose of this document* ... *1*

*Quick Summary* ... *2*

*Overview of the Anterior Cruciate Ligament (ACL) Treatment Pathway* ... *2*

*ACL injury management* ... *3*

*What is the current evidence for management?* ... *3*

*What do patients say?* ... *4*

- Phase 1 of the POP-ACLR study ... 4
- Overview ... 4
- Results ... 4
- Summary ... 6

*Why a preoperative intervention?* ... *8*

*What is the evidence for preoperative treatment in other orthopaedic surgeries?* ... *9*

*Reference List* ... *10*

## Research aims

The aim of this research is to develop an evidenced-based intervention to support adults (aged ≥18) after an Anterior Cruciate Ligament (ACL) injury and before surgery. The focus of the intervention is around information, decision making and treatment.

## Meeting aims

The consensus meeting(s) aim is to develop the intervention through a preliminary round of voting followed by group discussion(s) to agree the final components.

## Purpose of this document

This document outlines some key research to help us consider what the intervention might look like. It should take 15 minutes to read.

## Quick Summary

Optimal treatment before ACL surgery is unknown, current practice in the NHS varies.

The preoperative phase of treatment has been highlighted by patients as a period of uncertainty. Patient and public stakeholder events concluded that further research was warranted to determine optimal treatment for this period to improve outcomes and consistency of care.

Patients report the preoperative period to be challenging for a number of reasons. They experience difficulty with:

- Deciding upon injury management
- Navigating the breadth of ACL resources and deciding how generic information applies to them
- Understanding how best to prepare for surgery
- Interacting with and understanding what to expect of the treatment pathway for example, which healthcare professional(s) to contact, who is involved in their care, what realistic expectations of treatment are, how to best prepare for surgery and when they can expect to return to normal activities

## Overview of the Anterior Cruciate Ligament (ACL) Treatment Pathway

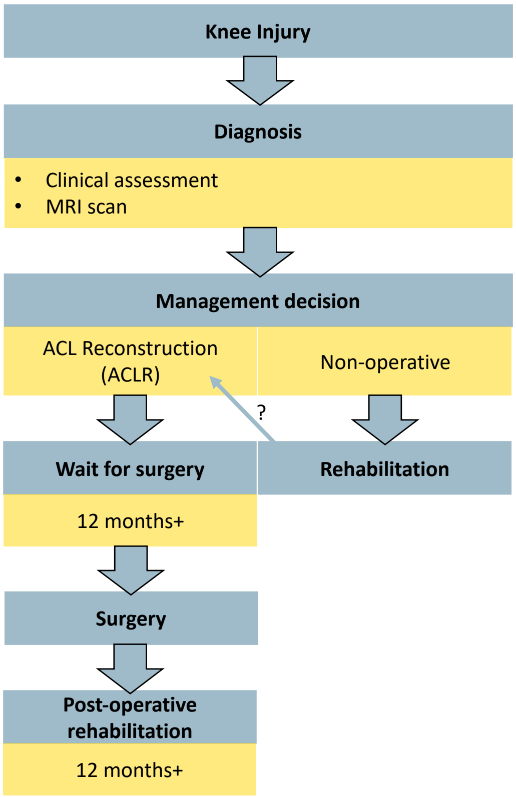


The above flow chart outlines a typical ACL injury pathway in the UK. Whilst this may vary between hospitals, this overview helps us to consider where the intervention may fit in.

## ACL injury management

Currently, there is no gold-standard way to make a decision about the treatment an individual should receive following an ACL rupture. Typically, surgery is recommended for those who wish to return to physical activity that involves cutting, pivoting and changing direction. In the UK, a large proportion of patients go on to have surgery (Anterior Cruciate Ligament Reconstruction, ACLR).

Whilst it is outside the scope of this consensus group to discuss and compare the treatment methods of surgery and non-operative management, it is important to consider this decision. Recent research (qualitative interviews; manuscript under development) has found that making decisions about surgery are difficult for patients, who perhaps need more support than previously thought. It was also found that individuals awaiting surgery were still uncertain whether this was the right treatment for them.

NHS England and the National Institute for Health and Care Excellence (NICE) recognise that shared decision making, between patients and healthcare professionals about treatment is important. For some conditions, such as knee and shoulder pain, decision support tools have been developed to help patients think through decisions about their condition and its management. You can view these here:

1) https://www.versusarthritis.org/about-arthritis/healthcare-professionals/musculoskeletal-decision-support-tools/
2) https://www.england.nhs.uk/personalisedcare/shared-decision-making/decision-support-tools/

## What is the current evidence for management?

The most recent, and only, randomised controlled trial (RCT) comparing surgery with rehabilitation in the UK was published in 2022.[1] The study included 315 participants and concluded that (at 18-months) those who received surgery had substantially better outcomes than those in the non-surgical group for:

- Self-reported knee outcomes (KOOS4 – a combination of questions about knee pain, symptoms, sport/recreational activity and knee-related quality of life)
- Quality-of-life (Anterior Cruciate Ligament Quality of Life Questionnaire)
- Activity level (Tegner score)

Of those who received rehabilitation, 41% went on to have surgery. Surgical reconstruction was also reported to be the most cost-effective treatment.

Two RCTs in other countries (Netherlands and Sweden) have also compared surgery against rehabilitation.[2,3] The Swedish study (2010) concluded that surgery was unnecessary for some and that rehabilitation should be trialled first and the Netherlands study (2021) suggested that early reconstruction may offer better results.

## What do patients say?

The POP-ACLR patient and public involvement group helped to design the study proposal, highlighting the preoperative phase of ACLR treatment as a period of uncertainty. They felt further research was warranted to explore treatment options for this period and to improve consistency in care.

### Phase 1 of the POP-ACLR study

Phase 1 of the POP-ACLR study interviewed ACL patients at three different time-points on the ACLR pathway in aim to understand:

(1) Patient experiences of treatment
(2) Views and involvement in prehabilitation
(3) Sources and consistency of healthcare advice.

The results from this study are currently being written up, please do not share these outside of this group.

### Overview

- 18 patients interviewed between August and November 2022
- Age
  - 18 to 45 (median age 29 years)
- Sex
  - 3 female
  - 15 male
- Ethnic origin (as described by the participant)
  - 14 White
  - 2 Indian
  - 1 British Asian
  - 1 Pakistani
- Time point on the pathway
  - 10 awaiting ACLR, 6 3-month post-surgery 2 1-year post-surgery
- Prehabilitation
  - 12 participants stated to have engaged in some sort of prehabilitation prior to surgery
- Activity level
  - 2 participants had returned to their preinjury level of physical activity, both were at the preoperative time-point.

### Results

Interview results were grouped into 5 themes. The table below shows these themes and a summary of what these represent. An example quote from interview transcripts has also been included for each theme.

| Theme | Summary of Theme Topics | Example Quote |
|---|---|---|
| 1) Injury experience, impact and support | • Varied experience of diagnosis<br>*e.g. multiple routes to diagnosis (GP, ED, Physio), missed-diagnosis*<br>• Physically and mentally challenging injury<br>• Difficulty with injury acceptance<br>• Burdensome and life-changing injury<br>• Existing support networks and support from those with injury experience is important | *"It's been **absolutely horrific** … there are days where I just lie in bed and I am just absolutely **heartbroken** because I can't do the things that I used to love doing?"*<br><br>*Pre-operative patient* |
| 2) Navigating the treatment pathway | • Pathway described as 'passive'<br>• Uncertainty around the decision to have surgery<br>• Limited support with decision making for surgery<br>• Lack of personalised care<br>• Communication with healthcare professionals is challenging | *When describing treatment, one patient explained: "you're just **going through the motions**"*<br><br>*Pre-operative patient* |
| 3) Sense making in the preoperative period | • Many unanswered questions and concerns<br>• Participants described lacking in confidence in a number of areas<br>• Prehabilitation (with physiotherapy) valued, participants suggested that it:<br>○ Offers advantage to recovery, postoperative rehabilitation and supports a faster return to physical activity<br>○ Supports psychological wellbeing<br>○ Provides increased knowledge of the injury and its management<br>○ Needs to be specific | *"For me, personally, doing the prehab has helped me **continue with daily activities**, so if I didn't do it, I probably would be a lot worse and I'm now in a **better position to have surgery** because my tissue's healing well"*<br><br>*Pre-operative patient* |
| 4) Uncertainty, expectations and reality of the post-surgical period | • Inpatient environment is overwhelming with delays experienced and interactions limited<br>• The first few weeks following surgery are a particularly challenging time<br>• Mental wellbeing is a reflection of physical progress<br>*e.g. as physical function improves so does mental wellbeing*<br>• Range of expectations regarding return to work and physical activity<br>○ Limited expectation management | *"it's about **two weeks you don't see anybody about it** … you're expected to crack on with the exercises, in a lot of pain aggravation you can imagine all the thoughts going through your mind should I have the surgery all of these things, and all you're left is really a **booklet** to read so **doesn't really take on our thoughts and feelings** about what is happening there and then."*<br><br>*3-month postoperative patient* |

| | | |
|---|---|---|
| 5) Balancing resources, advice and opinions | • Resources are difficult to navigate and thought to be unreliable<br>• Standardised information is not well received<br>• Absent information on surgery, how to prepare for it and what to expect after it<br>• Information regarding pathway timelines was a common frustration among participants who described to feel "in the dark"<br>• Media influential on treatment expectations<br>• Conflicting advice - physiotherapists, surgeons, friends, family members, colleagues<br>• Cultural influences on treatment expectations and outcomes | *"the information is everywhere, **I don't know which one I should believe** and which one I shouldn't believe"*<br><br>*Pre-operative patient* |

These results represent the challenges patients face when diagnosed with an ACL injury and the difficulties of navigating the NHS treatment pathway.

## Summary

Key findings from this study include:

- **Lack of consistent and reliable information** regarding the injury and its treatment

- **Lack of support with decision making** regarding treatment, this resulted in patients feeling helpless. Limited demonstration of shared decision making:
    - Some explained to have not been involved in the decision to have surgery
    - Some did not want to feel responsible for the decision regarding surgery due to fear of making a 'wrong' decision
    - Some felt they were presented with no choice but to have surgery as if they didn't, this would limit lifelong engagement in physical activity
    - Patients wanted a recommendation from their healthcare professional which was not always offered

- **Guidance on how to manage the condition whilst awaiting surgery was limited.** Participants where unclear which healthcare professional to contact for support, what to expect in the preoperative period and how they could best prepare for surgery

- **Prehabilitation was valued** highly amongst participants, they felt it offered an advantage to:
    - Recovery and postoperative rehabilitation
    - Returning to physical activity/sport faster
    - Psychological/mental wellbeing and increased knowledge of the injury and its management
    - Support decision making for surgery (e.g. exhaust prehabilitation first before deciding to proceed with surgery)

- **Specificity of prehabilitation was important**, standard and generalised exercise prescription was not well received. Where prehabilitation was 'general' this was deemed low value and resulted in reduced engagement from the participant

- **Standardised information** that was not personalised/tailored to the participant was **not well received**

- **Healthcare advice was inconsistent** and received from a range of individuals including healthcare professionals (such as surgeons, physiotherapists, nurses and general practitioners), friends, family members and colleagues

- **Communication with and between healthcare professionals was disjointed**. Participants explained to have little time during consultations at various points on the pathway (e.g. initial diagnosis, appointments with orthopaedics regarding surgery, inpatient ward) which left them feeling rushed and overwhelmed. Patients were also frustrated when healthcare professionals did not liaise with each other regarding their care

## Why a preoperative intervention?

The wait for an ACLR in the UK is over 12-months. Optimum treatment prior to surgery is unknown.[4,5] Previous evidence has suggested that treatment should address both physical and mental health but little further is described as to specific treatment approaches.

A 2020 systematic review explored the effectiveness of preoperative rehabilitation programmes (commonly referred to as 'prehabilitation' or 'prehab') on post-operative outcomes.[5] The evidence was limited, with only three RCTs relevant for inclusion. The intervention in all three studies was a rehabilitation programme, however these varied in content, frequency and length. The outcomes used to evaluate the effects of the prehab programme were muscle strength, function, pain and return to sport. Results concluded that prehabilitation offers some benefits to quadriceps strength and single leg hop distance three months after ACLR.

As there is little guidance in the literature as to what prehabilitation should entail, a 2021 survey explored physiotherapy practice for ACL patients waiting for surgery.[4] Although advice, education and exercise were cited as consistent components of treatment, the types of exercises given, how often exercises should be completed and the length of treatment varied. A range of other interventions were also reported (e.g. manual therapy, electrotherapy, bracing), confirming that prehabilitation varies. A 2022 review confirmed that there this is little consensus on what should be included in ACL rehabilitation (both prior to and after surgery) leading to a variety of protocols used in practice.[6]

It has previously been reported that some preoperative factors can predict post-operative outcomes. A 2023 systematic review (results pending publication, please do not share) aimed to identify modifiable factors from the preoperative period that may influence return to physical activity outcomes after ACLR. The review found four factors to be associated with a return to physical activity after ACLR:

1) Quadriceps strength (higher strength)
2) Psychovitality – a questionnaire designed to assess patients' expectations related to treatment outcome and motivation to resume pre-injury activity levels (score ≥15)
3) Positive estimation of ability to return to preinjury level
4) Quadriceps tendon graft (Bone-Patella Tendon-Bone)

These results can help us to consider what factors may be beneficial to target in ACL patients awaiting surgery.

## What is the evidence for preoperative treatment in other orthopaedic surgeries?

NICE guidelines recommend that prehabilitation is offered to patients awaiting total hip and knee replacement surgery.[7] NICE suggest that this should include:

- Exercises
- Lifestyle advice including weight management, diet and smoking cessation
- Support to maximise functional independence and quality of life

A 2023 systematic review and meta-analysis explored the effectiveness of prehabilitation in all orthopaedic surgeries.[8] There were 48 studies included covering total knee replacement, total hip replacement, lumbar spine, ACL and hip impingement surgery (only one study included ACL patients and this study was included in the 2020 review mentioned above). 81% of the prehabilitation programmes used exercise interventions and only 10% had multiple components to the intervention. The table below shows the results of the review and the benefits of prehabilitation in the different patient populations.

| | | Total Knee Replacement | Total Hip Replacement | Lumbar Spine Surgery | Hip impingement surgery | ACL |
|---|---|---|---|---|---|---|
| Preoperative | Reduced pain | ✓ | ✓ | ✓ | | |
| | Improved function | ✓ | ✓ | ✓ | | |
| | Improved health-related quality of life | | ✓ | ✓ | | |
| | Improved muscle strength | ✓ (inner thigh muscles – adductors) | ✓ (front thigh muscles – knee flexors) | | | |
| Postoperative | Reduced pain | | | ✓ | | |
| | Improved function | ✓ 6-weeks & 3-months | ✓ 3-months and 12-months | ✓ 6-months | | |
| | Improved health-related quality of life | ✓ 6-weeks & 3-months | | | | |
| | Improved muscle strength | ✓ (front and back thigh muscles - knee flexors and extensors) | | | | |

## Reference List

1. Beard, D. J. *et al.* Rehabilitation versus surgical reconstruction for non-acute anterior cruciate ligament injury (ACL SNNAP): a pragmatic randomised controlled trial. *The Lancet* **400**, 605–615 (2022).
2. Reijman, M. *et al.* Early surgical reconstruction versus rehabilitation with elective delayed reconstruction for patients with anterior cruciate ligament rupture: COMPARE randomised controlled trial. *The BMJ* **372**, (2021).
3. Frobell, R. B., Roos, E. M., Roos, H. P., Ranstam, J. & Lohmander, L. S. A Randomized Trial of Treatment for Acute Anterior Cruciate Ligament Tears. *New England Journal of Medicine* **363**, 331–342 (2010).
4. Carter, H. M., Webster, K. E. & Smith, B. E. Current preoperative physiotherapy management strategies for patients awaiting Anterior Cruciate Ligament Reconstruction (ACLR): A worldwide survey of physiotherapy practice. *Knee* **28**, 300–310 (2021).
5. Carter, H. M., Littlewood, C., Webster, K. E. & Smith, B. E. The effectiveness of preoperative rehabilitation programmes on postoperative outcomes following anterior cruciate ligament (ACL) reconstruction: A systematic review. *BMC Musculoskelet Disord* **21**, 1–13 (2020).
6. Culvenor, A. G. *et al.* Rehabilitation after anterior cruciate ligament and meniscal injuries: a best-evidence synthesis of systematic reviews for the OPTIKNEE consensus. *Br J Sports Med* **0**, 1–10 (2022).
7. NICE. Recommendations | Joint replacement (primary): hip, knee and shoulder | Guidance | NICE. (2020).
8. Punnoose, A. *et al.* Prehabilitation for Patients Undergoing Orthopedic Surgery: A Systematic Review and Meta-analysis. *JAMA Netw Open* **6**, e238050–e238050 (2023).
