# Supplementary material for: Development of an intervention for patients following an anterior cruciate ligament rupture: an online nominal group technique consensus study
Source: BMJ Open. 2024 Jul 18;14(7):e082387. doi: 10.1136/bmjopen-2023-082387 (PMC11261705; doi:10.1136/bmjopen-2023-082387)
Supplement: online supplemental file 3 [file bmjopen-14-7-s003.pdf]

POP-ACLR Pre-Meeting Voting

Below is a list of components for consideration in the preoperative treatment package/intervention. Please select one answer to indicate how important you think each component is for inclusion.

Component 1

Advice and Education.

1. Preoperative advice and education should include:

	Very important	Important	Neither important nor not important	Not important	Not at all important
Knee anatomy and injury	○	○	○	○	○
Surgical procedure	○	○	○	○	○
Evidence-based information regarding graft choice	○	○	○	○	○
Risks of surgery	○	○	○	○	○
ACL injury management - surgery versus rehabilitation	○	○	○	○	○
Pathway milestones, expected appointments and timeframes e.g. physiotherapy, surgery, orthopaedic follow-up	○	○	○	○	○
Evidence based information on injury outcomes e.g. re-rupture, return to work, return to physical activity	○	○	○	○	○
Expected timeline specific to the patient e.g. considering prognostic factors that may delay a return to physical activity/work	○	○	○	○	○
Evidence-based information					

[illegible] regarding outcomes of surgical and non-surgical treatment	○	○	○	○	○
Prehabilitations e.g. purpose, importance, timeframe	○	○	○	○	○
Discussing the postoperative period	○	○	○	○	○
What to expect during the inpatient stay	○	○	○	○	○
Common issues pre- and post-surgery	○	○	○	○	○
Pain management	○	○	○	○	○
Managing work and home preparations e.g. expected absence from work, expectations for return to work, childcare, driving	○	○	○	○	○
Common psychological difficulties associated with ACL injury	○	○	○	○	○
Signposting towards relevant services for mental health support	○	○	○	○	○
Weight management and nutrition	○	○	○	○	○
Smoking cessation	○	○	○	○	○
Goal setting	○	○	○	○	○

2. Free text for any further comments you wish to make with regards to advice and education

Component 2

Exercise Guidance

3. Exercise guidance in the preoperative period should include:

	Very important	Important	Neither important nor not important	Not important	Not at all important
At least 1 session with a rehabilitation/exercise therapist e.g. physiotherapist, sports rehabilitator	◯	◯	◯	◯	◯
2-6 sessions with a rehabilitation/exercise therapist e.g. physiotherapist, sports rehabilitator	◯	◯	◯	◯	◯
More than 6 sessions with a rehabilitation/exercise therapist e.g. physiotherapist, sports rehabilitator	◯	◯	◯	◯	◯
Exercise type(s)	◯	◯	◯	◯	◯
Frequency of exercise completion	◯	◯	◯	◯	◯
Exercise programme length	◯	◯	◯	◯	◯
Time-period to be completed e.g. immediately after injury, 3-months prior to surgery, continuous	◯	◯	◯	◯	◯
Delivery method e.g. virtual, group, individual, guided by a healthcare professional, self-directed	◯	◯	◯	◯	◯
Guidance on exercise/ types of activity to avoid	◯	◯	◯	◯	◯

4. Free text for any further comments you wish to make with regards to exercise guidance or delivery method

Component 3

Delivery method

5. The preoperative intervention should be delivered via:

	Very important	Important	Neither important nor not important	Not important	Not at all important
Booklet	◯	◯	◯	◯	◯
Website	◯	◯	◯	◯	◯
Face-to-face	◯	◯	◯	◯	◯
Forum	◯	◯	◯	◯	◯
Group	◯	◯	◯	◯	◯
1:1	◯	◯	◯	◯	◯
Peer support	◯	◯	◯	◯	◯
By a single healthcare profession	◯	◯	◯	◯	◯
By a team of healthcare professionals	◯	◯	◯	◯	◯
Combination of face-to-face interactions and printed/ digital resources	◯	◯	◯	◯	◯

6. Free text for any further comments you wish to make with regards to intervention delivery method

Component 4

Outcome measures

7. Outcome measures to be collected/used as part of the preoperative intervention should include:

	Very important	Important	Neither important nor not important	Not important	Not at all important
Knee-specific outcome score e.g. Knee injury and Osteoarthritis Outcome Score (KOOS)	○	○	○	○	○
Psychological measures e.g. anxiety and depression screening	○	○	○	○	○
Clinical assessment e.g. knee range of motion, muscle strength	○	○	○	○	○
Weight screening	○	○	○	○	○
Patient satisfaction	○	○	○	○	○
Pre-injury level of activity	○	○	○	○	○
Current level of activity	○	○	○	○	○
Expectation screening	○	○	○	○	○
Patient estimation of ability to return to preinjury activity levels	○	○	○	○	○
Occupation	○	○	○	○	○

8. Free text for any further comments you wish to make with regards to outcome measures

Component 5

Decision Making

9. Decision-making support should be:

	Very important	Important	Neither important nor not important	Not important	Not at all important
Primarily for the patient in the form of an information sheet with interactive components for them to bring to consultations e.g. sections to fill in / questions to respond to	○	○	○	○	○
For both the healthcare professional and the patient to complete together	○	○	○	○	○
In the form of a paper document	○	○	○	○	○
In the form of an online tool	○	○	○	○	○
Both a paper and online tool	○	○	○	○	○
A flow-chart of key questions to help consider treatment options	○	○	○	○	○

10. Decision-making support include:

	Very important	Important	Neither important nor not important	Not important	Not at all important
Education on the condition					
Treatment options					
Patient preference for treatment					
Clinician preference / recommendation for treatment					
Space for the patient to document/consider what is important to them about the recovery of their knee injury e.g. reduced pain, improved mobility, return to work					
Space for the patient to document/consider what is important to them in their treatment					
Information about rehabilitation					
Information about surgery					
Space for the patient to document/consider how they feel about their treatment options					
Support to make a decision about treatment					
Resource signposting					
Up-to-date and evidence-based outcome information e.g. percentage of those who return to physical activity following surgery					

11. Free text for any further comments you wish to make with regards to decision making.

This content is neither created nor endorsed by Microsoft. The data you submit will be sent to the form owner.

Microsoft Forms
