# Supplementary material for: Development of an intervention for patients following an anterior cruciate ligament rupture: an online nominal group technique consensus study
Source: BMJ Open. 2024 Jul 18;14(7):e082387. doi: 10.1136/bmjopen-2023-082387 (PMC11261705; doi:10.1136/bmjopen-2023-082387)
Supplement: online supplemental file 4 [file bmjopen-14-7-s004.pdf]

POP-ACLR Pre-Meeting Voting 2

Advice and Education

This section is related to the advice and education recommendations. The file detailing these is attached to the email along with this survey link.

1. Based on the advice & education recommendations – are you happy these represent what was discussed?

   ○ Yes

   ○ No

2. Do you feel there is anything to add to the advice and education recommendations, or is there anything you feel is missing? Any other comments you would like to make, please note them here.

Exercise Guidance

This section is related to the exercise recommendations. The file detailing these is attached to the email along with this survey link.

3. Based on the exercise recommendations – are you happy these represent what was discussed?

◯ Yes

◯ No

4. Do you feel there is anything to add to the exercise recommendations, or is there anything you feel is missing? Any other comments you would like to make, please note them here.

5. During the meeting, a number of different 'types' of exercise were suggested and/or discussed. Thinking about what you think should be included **as a minimum**, please select the three types you think are most important.

Please select at most 3 options.

☐ Strength

☐ Mobility

☐ Proprioception

☐ Impact/plyometric

☐ Normal sporting/physical activity

☐ Cutting/pivoting

☐ Cardiovascular exercise

☐ Inflammation / swelling management

☐ Vocational rehabilitation to support return to work

☐ Other

6. During the meeting, we voted on & discussed some things we may consider suggesting ACL patients avoid prior to surgery. We eluded to the idea that perhaps nothing should be avoided. Reading the recommendations of Do's and Don'ts - do you agree with this statement?

☐ Yes

☐ No

7. Anything else you would like to add for 'Do's and Don'ts' or any other comments you would like to make?

8. Do you think exercise guidance should differ for those who have decided to have surgery versus those who have opted for non-operative management?

- ◯ Yes
- ◯ No

9. If yes to question 8 - why?

Delivery Method

This section is related to the delivery method recommendations. The file detailing these is attached to the email along with this survey link.

10. Based on the delivery method recommendations – are you happy these represent what was discussed?

- ◯ Yes
- ◯ No

11. Do you feel there is anything to add to the delivery recommendations, or is there anything you feel is missing? Any other comments you would like to make, please note them here.

Outcome Measures

This section is related to the outcome measure recommendations. The file detailing these is attached to the email along with this survey link.

12. Do you think outcome measures used should differ for those who have decided to have surgery versus those who have opted for non-operative management?

◯ Yes

◯ No

13. If yes to Question 12, why? What do you think should differ?

14. Based on the previous voting and discussions, 3 outcome measures were considered very important to be used, at a minimum, with every patient. These included:

    1. Knee specific outcome measures
    2. Psychological outcome measure
    3. Current level of activity including occupation

    Are there any specific outcome measures you feel should be included under these categories, for example the Knee injury and Osteoarthritis Outcome Score (KOOS) or Tegner activity level.

Shared Decision Making

This section is related to the shared decision making recommendations and tool. The file detailing these is attached to the email along with this survey link.

15. Based on the shared decision making recommendations – are you happy these represent what was discussed?

◯ Yes

◯ No

16. Do you feel there is anything to add to the shared decision making recommendations, or is there anything you feel is missing? Any other comments you would like to make, please note them here.

17. The shared decision making tool is split into 3 components which we will discuss in more detail at meeting 2. The 3 documents are attached to your email and include:

    1. Patient information leaflet
    2. 'What should I be thinking about' diagram
    3. Option grid

    Ahead of the meeting, it would be helpful if you could review these & make any comments in the following text box. For example, is there anything missing? Is it too short/too lengthy? Any suggestions for change?

This content is neither created nor endorsed by Microsoft. The data you submit will be sent to the form owner.

Microsoft Forms
