# Supplementary material for: Development of an intervention for patients following an anterior cruciate ligament rupture: an online nominal group technique consensus study
Source: BMJ Open. 2024 Jul 18;14(7):e082387. doi: 10.1136/bmjopen-2023-082387 (PMC11261705; doi:10.1136/bmjopen-2023-082387)
Supplement: online supplemental file 6 [file bmjopen-14-7-s006.pdf]

Making a Decision about Anterior Cruciate Ligament Injury Treatment

This option grid presents evidence-based information about treatment for an anterior cruciate ligament (ACL) rupture. You should use this with your healthcare professional to help you make the right decision for you.

	**Surgery (ACL reconstruction)**	**Rehabilitation (non-operative management)**
**What are my options?**	Surgery involves reconstructing your torn ACL with a graft taken from another part of your body (typically a tendon from your hamstring or knee). Sometimes a donor graft may be used.	Rehabilitation involves working with a therapist. The areas targeted during treatment are specific to you and your injury but typically aim to improve your overall fitness, knee function, strength and stability.
**What do I need to know about this treatment?**	Surgery involves a keyhole operation to reconstruct your torn ACL with a graft. This takes approximately 1 hour and will require you to be under local or general anesthetic.<br><br>You may need to wear a brace after surgery and use elbow crutches.	Rehabilitation requires you to commit time to a regular exercise programme. This may be in a one-to-one or group setting. Your therapy sessions are unlikely to be supervised for the duration of your treatment and you will need to complete your exercises independently at home or in a gym.
**What does the research say about this treatment option?**	A trial completed in the UK (2022) reported surgery to be better than rehabilitation for patients with an ACL tear older than 4-months. In this trial, it was concluded that those who had surgery had better scores 18-months after surgery for knee outcomes, quality of life and activity level.<br><br>However, 2 previous trials have concluded that:<br>▪ surgery may be unnecessary for some and rehabilitation should be trialed first<br>▪ early reconstruction may be better than delayed reconstruction<br><br>Both trials were completed outside the UK in different healthcare settings.	The UK 2022 trial reported that 41% of patients went on to have surgery after a period of rehabilitation.<br><br>There has previously been no consensus on the best rehabilitation to offer to patients. Because of this treatment has varied across the UK and in research studies. In 2023 a group of patients and clinicians agreed some recommendations for rehabilitation to help improve and standardise treatment.<br><br>We know that some patients manage well without surgery. At present (2023) it is unclear who is an ideal candidate for surgery and who may manage without it.
**What are the outcomes of this treatment?**	**Preinjury activity level** - 20% of patients return to their preinjury levels of activity 1-year after surgery, 28% return at 18-months and less than 45% return at 2-years.<br><br>**Work** – The average time to return to work after ACL reconstruction is approximately 2 and a half months. This may be shorter for desk-based jobs and longer for more labour-intensive roles.<br><br>**Driving** – Typically you can return to driving 6-weeks after your operation.	**Preinjury activity level** - 24% of patients return to their preinjury activity level 18-months after surgery.<br><br>**Work** – no research to date has collected information on the time to return to work following ACL injury for patients who chose non-operative treatment.<br><br>**Driving** – there is no set restriction on returning to driving after an ACL rupture. This should be decided by you and your clinician.
**If I choose this treatment, what happens next?**	If you decide to have surgery, you will be placed on the waiting list. Your clinician should be able to give you an estimated waiting time. Your surgeon will also discuss your graft choice with you. You should also be referred to physiotherapy for support whilst you wait for surgery.	You will be referred to physiotherapy, your clinician should be able to give you an estimated waiting time.<br><br>You will work with your physiotherapist to develop some goals to work towards. You should start thinking about these now, ready to discuss at your first appointment.

<table>
<tr><td>What are the risks?</td><td>The risks associated with surgery include:
▪ Re-rupture (7.8%)
▪ Cyclops lesion (21-35%)
▪ Infection (less than 2%)
▪ Blood clot (less than 1%)
▪ Donor site pain/discomfort
▪ Numbness around the knee

There is no increased risk of osteoarthritis should you decide to have surgery.</td><td>The risks associated with rehabilitation include:
▪ Ongoing, symptomatic instability
▪ Potential for further injury (unclear risk)

It is a common thought that, without surgery, you are at risk of a meniscal (cartilage) injury. There is evidence to suggest this may or may not be true. As of 2023, the evidence is unclear.

There is no increased risk of osteoarthritis should you decide not to have surgery.</td></tr>
<tr><td>Will I need to have more treatment?</td><td>After your ACL reconstruction you will need to undergo a progressive rehabilitation programme with a physiotherapist. This will take at least 6-months and depending on your goal (e.g. if you wish to return to competitive sports), may take longer than 12-months.

If complications arise, further surgery may be needed. Otherwise, the graft remains in place and does not need to be replaced in the future unless you have a second injury.</td><td>If you do not return to your desired functional level, you can discuss the option of surgery with your clinician.</td></tr>
<tr><td>Are there any other options?</td><td>Repair
There is limited evidence that ACL repair is a successful surgical option to treat an ACL rupture. ACL repair is a technique that does not use a graft and instead repairs the torn ligament with stitches.

Research in this area is limited and so at present (2023), we do not have enough evidence to suggest the success of this treatment.</td><td>Self-healing
New evidence was published in 2022 showing the ACL to have healed (to some extent) without surgery. This has been reported in a small number of patients and the long-term effects are mostly unknown.

Research in this area is limited and so at present (2023), we do not have enough evidence to suggest the success of this treatment.</td></tr>
<tr><td>What else can I do?</td><td colspan="2">It is important that you consider the following:
▪ Fitness management – your clinician may discuss your overall health and fitness with you. This may include your cardiovascular health and weight management to support you in the recovery of your ACL rupture and/or help you prepare for surgery.

▪ Psychological factors – research suggests that psychological barriers, such as fear of reinjury, are the most common reason for patients failing to return to their preinjury level of activity. There is also research to suggest that some psychological factors, measured before surgery, can predict your return to preinjury levels after surgery. If you are concerned about your mental wellbeing and feel you would like further support, talk to your clinician. They can support you to access further help if needed.

▪ Optimising your sleep and diet/nutrition may also be important to help your recovery, if you would like further support, talk to your clinician.

▪ Take time to think about what your end goal is, this will help you to think about whether surgery or rehabilitation is the best option for you.</td></tr>
<tr><td>What does my clinician recommend?</td><td colspan="2">You may wish to gain a recommendation from more than one clinician.
(e.g. surgeon 1, physiotherapist, surgeon 2)</td></tr>
<tr><td>What is my treatment preference?</td><td colspan="2">It is important to think about what treatment you think you would like to have. Write this here to discuss with your clinician.</td></tr>
</table>
