# Supplementary material for: Development of an intervention for patients following an anterior cruciate ligament rupture: an online nominal group technique consensus study
Source: BMJ Open. 2024 Jul 18;14(7):e082387. doi: 10.1136/bmjopen-2023-082387 (PMC11261705; doi:10.1136/bmjopen-2023-082387)
Supplement: online supplemental file 7 [file bmjopen-14-7-s007.pdf]

Post-injury/pre-operative recommendations for patients following anterior cruciate ligament (ACL) rupture

This document provides recommendations for patients following an ACL rupture who may be awaiting surgery or proceeding with non-surgical management. The recommendations include:

- Advice and education
- Exercise guidance
- Delivery method
- Outcome measure use
- Shared decision making

The recommendations were developed using the nominal group technique (NGT) approach involving a group of patients and professionals in the UK. The group consisted of the lead researcher, expert facilitators (patients, clinical and academic experts) and research participants including:

- 2 patients
- 4 therapists [physiotherapists and occupational therapist]
- 1 surgeon
- 1 MSK outpatient therapy manager

A 70% threshold was used to determine consensus on component items. Items were voted and discussed to be considered as 'very important', 'important', 'neither important nor not important', 'not important' or 'not at all important'. Our recommendations are based on those that were considered to be '**very important**' and '**important**' amongst the group.

FUNDED BY NIHR | National Institute for Health and Care Research
POP-ACLR
NHS University Hospitals of Derby and Burton NHS Foundation Trust
University of Nottingham UK | CHINA | MALAYSIA

Advice and Education Recommendations

5 advice and education items were considered to be **very important**. We recommend these items as the **minimum elements** to cover when delivering advice and education to patients following diagnosis of an ACL rupture. As a minimum, post-injury/pre-operative ACL advice and education **should include**:

1. Advice on injury management
   e.g., surgery or rehabilitation
2. Evidence based information on injury outcomes
   e.g., information about re-rupture, return-to-work, return to physical activity/sport
3. Expected timeline, specific to the patient, from present to return to patients end goal e.g. return to sport
   e.g., return to sport
4. Information about pathway milestones including expected appointments and timeframes
5. Evidence based info on outcomes of treatment

We recommend the following **16 items**, considered to be **important**, to be included at the discretion of the clinician, to tailor advice/education to each individual patient. Topics **to be considered** include:

- Knee anatomy and injury
- Surgical procedure
- Risks of surgery
- Prehabilitation
- Goal setting
- Smoking cessation
- Common issues pre- and post-operation
- Pain management
- Weight management & nutrition
- Evidence based information regarding graft choice
- What to expect during the inpatient stay
- Postoperative period
- Common psychological difficulties associated with ACL injury
- Signposting to relevant services for mental health support
- Managing work and home preparations
- Information regarding spontaneous ACL healing

Advice and Education Recommendations

Whilst signposting to relevant services for mental health support is not included as a very important item, the group emphasise that this should be considered by clinicians for all patients following ACL injury. The extent to which this is discussed, and the amount of support offered however, must be **tailored to each individual patient**.

We recommend advice and education to be delivered **at the point of injury diagnosis** (or shortly after) **before a definitive decision about treatment** is made.

It is further essential to ensure the advice and education delivered is **consistent** throughout the patients care. Although we did not feel it was important to distinguish who (i.e. which profession) should deliver the advice/education, we recommend that it is delivered in a **one-to-one consultation**.

In summary, advice and education **should be delivered**:

- In a **one-to-one** session
- By **any clinician** with expertise and knowledge to do so
- **Consistently** by the healthcare team across the patients care

Exercise Recommendations

A range of components were discussed in relation to exercise guidance including:

1. Number of sessions
2. Exercise type
3. Exercise programme length and frequency
4. Risks and considerations

1. Number of sessions

It was considered to be **very important** that, as a minimum:

At least one-session to be offered with a rehabilitation / exercise therapist, **within 3-months** of ACL injury diagnosis

We recommend that clinicians determine individual patients' need and offer further sessions, where it is deemed appropriate considering **specific patient goals and waiting times** for surgery. Where more than 6 sessions are offered, goals need to be revisited and rehabilitation progressed to support patient motivation and engagement.

2. Exercise type

We recommend that clinicians **consider the appropriateness** of the following types of exercise in a programme for patients following ACL injury:

- Strength
- Mobility
- Proprioception
- Vocational rehabilitation
- Impact and plyometric
- Normal sporting activities
- Inflammation and swelling management

Selection needs to be **specific** to each patient and based on their **goals**.

Exercise Recommendations

3. Exercise programme

It was considered **very important** that, as a **minimum**, exercise programme's:

- Include a variety of exercise types (including the 3 very important types)
- Be completed **2-3 times a week**
- Be completed **continuously** by the patient for the duration of the preoperative period (not to be supervised for the entirety of the preoperative period)

4. Risks and considerations

It was considered **very important** that:

The **risks** of engaging in physical activity are discussed with each patient.

This discussion should include a balanced presentation of the evidence considering potential **risks and benefits**.

We recommend that exercise is guided by the appropriate professional and should be **within the limits of the individual**. Whilst it is acknowledged that activities that provoke knee swelling and instability and and cause intolerable amounts of pain may need to be **limited**, we recommend **progressive and graded exposure** to activities, working towards the patient's individual goals.

The risk benefit discussion should be based on the patients' goals to allow them to make educated decisions about engaging in physical activity after their ACL injury. The group discussed the importance of this discussion in **empowering patients** to engage in physical activity within the **scope of risk deemed acceptable to** them and their treating clinician(s).

For patients proceeding with surgery, consider the risks of further injury and the subsequent consequences to the planned surgical procedure. **Caution** to return to cutting/pivoting activities **may be greater** with this patient group.

Delivery Method Recommendations

The delivery method for any intervention was discussed amongst the group with the following **6 mediums** considered **important**.

- Booklet
- Website
- Face-to-face
- One-to-one
- Peer support
- Combination of face-to-face and digital or printed resources

We discussed the importance of the delivery method meeting the needs of the individual patient. Clinician's should consider the trade-off between a 1:1 session that offers tailored support and the peer support offered in a group setting.

We recommend an **initial 1:1 session** to ensure specific needs are met. The above methods should be utilised as appropriate for individual patients.

Outcome Measure Recommendations

3 outcome measures were considered to be **very important** for use with patients following ACL injury. As a minimum, we recommend clinicians **should** record:

- Knee specific outcome measure
- Psychological outcome measure
- Current level of activity including occupation

The following **6** measures were considered to be **important** and are advised to be used at the discretion of the clinician, selected as appropriate to each patient. Outcome measures **to be considered** include:

- Patient estimation of ability to return to their preinjury level
- Expectation screening
- Preinjury level of activity
- Patient satisfaction
- Weight screening
- Clinical assessment e.g. knee range-of-motion, leg strength

We discussed the need to alter outcome measure use according to the patients decision to proceed with surgical or non-surgical management. The group agreed that outcome measure use should not differ. We encourage clinicians to consider assessing patients at baseline regardless of their treatment decision as this may help with future treatment planning and progress tracking.

Shared Decision Making Recommendations

6 principles were considered to be **very important** to include in a shared decision making tool / conversation for patients following ACL injury. At a minimum, shared decision making **should** include:

- Up-to-date evidence-based outcome information e.g. percentage of those who return to preinjury levels of surgery
- Education on the condition
- Treatment options
- Risks
- Clinician preference and recommendations
- Patient preferences for treatment

The following 7 principles were considered to be **important** and are advised to be included at the discretion of the clinician/those developing the tool. Shared decision making principles **to be considered** include:

- Space for the patient to document / consider how they feel about their treatment options
- Space for the patient to document/consider what is important to them in their treatment
- Space for the patient to document/consider what is important to them about the recovery of their knee injury
- Support to make a decision about treatment
- Information about rehabilitation
- Information about surgery
- Resource signposting

It was also **considered important** that a shared decision making tool:

- Is for the healthcare professional and patient to complete together
- Is available online and paper
- Includes a flow chart of key questions to help the patient consider their treatment options

Recommendations Summary 1

Advice and education should:

1

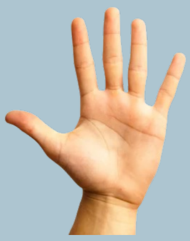

Include the 5 **very important** elements

1. Injury management
2. Injury outcomes
3. Treatment outcomes
4. Expected timeline
5. Pathway milestones

2

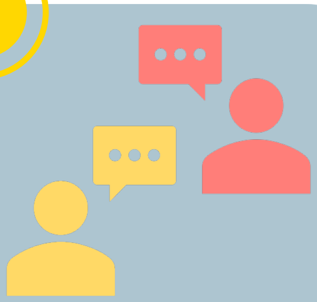

Be delivered in a **one-to-one** session by a clinician with appropriate **knowledge** and **expertise**

3

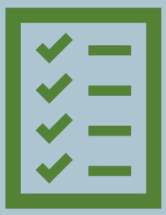

Be **consistent** across the patients care

Exercise should:

1

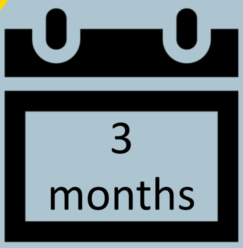

Include at least **1 session** within 3-months of diagnosis

2

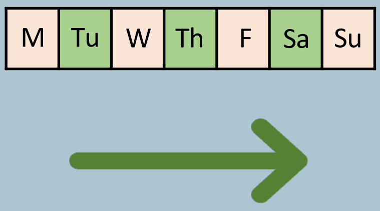

Be completed **2-3 times a week** and **continuously** whilst awaiting surgery

3

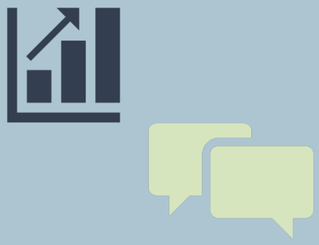

Be **graded** and **progressive** ensuring a **risk discussion** is had with every patient

Recommendations Summary 2

Intervention delivery:

1

Should include an initial **1:1 session**

2

Could be delivered via several **different mediums**

Outcome measures should include:

1

Knee specific measure

2

Psychological measure

3

Current **activity** level including **occupation**

Recommendations Summary 3

Shared decision making should include:

1. **Outcome** information
2. **Education** on the condition
3. **Treatment** options
4. **Risks**
5. **Clinician preference** & recommendation
6. **Patient preferences** for treatment
