# Supplementary material for: Development of an intervention for patients following an anterior cruciate ligament rupture: an online nominal group technique consensus study
Source: BMJ Open. 2024 Jul 18;14(7):e082387. doi: 10.1136/bmjopen-2023-082387 (PMC11261705; doi:10.1136/bmjopen-2023-082387)

Supplementary File 8 – Use of guidelines and supporting theory

Elwyn G, O'Connor A, Stacey D, *et al.* Developing a quality criteria framework for patient decision aids: online international Delphi consensus process. *BMJ*. 2006;333:417–9

Quality Criterion Framework	Development of the shared decision-making intervention
Systematic development process	Nominal group technique consensus study.
Providing information about options	Included in the patient information leaflet and option grid.
Presenting probabilities	Probabilities included where available for each treatment option, this is presented in both the patient information leaflet and option grid.
Clarifying and expressing values	Section on the option grid included for patient treatment preferences.
Using patient stories	Patient experiences are embedded into the design of the intervention. Specific stories were not included however the section 'What else can I do?' of the option grid was driven by patient feedback of things they felt were important for patients to know.
Guiding or coaching in deliberation and communication	Prompts included in the intervention for both patients and clinicians. The intervention is planned to be delivered with clinicians who will receive training on its use with patients.
Disclosing conflicts of interest	Included on the first page of the patient information leaflet.
Delivering patient decision aids on the internet	During feasibility testing the material will be made available online.
Balancing the presentation of options	Embedded into the design and feedback to be gained from patients during feasibility testing regarding the presentation of the treatment options.
Using plain language	We have endeavoured to use plain language throughout the intervention, checking this with the patient participants in the study. We will also gain feedback on readability/language used in the intervention during feasibility testing.
Basing information on up to date scientific evidence	Up-to-date evidence used and referenced throughout.
Establishing effectiveness	Funding gained for feasibility testing ahead of testing in a future large main trail.

Adaptation of Figure 1 – Model Development Process for Decision Aids from Coulter A, Stilwell D, Kryworuchko J, *et al.* A systematic development process for patient decision aids. *BMC Med Inform Decis Mak*. 2013;13 Suppl 2. doi: 10.1186/1472-6947-13-S2-S2.

Text in black represents guidance from Coulter et al., text in blue demonstrates steps taken intervention development

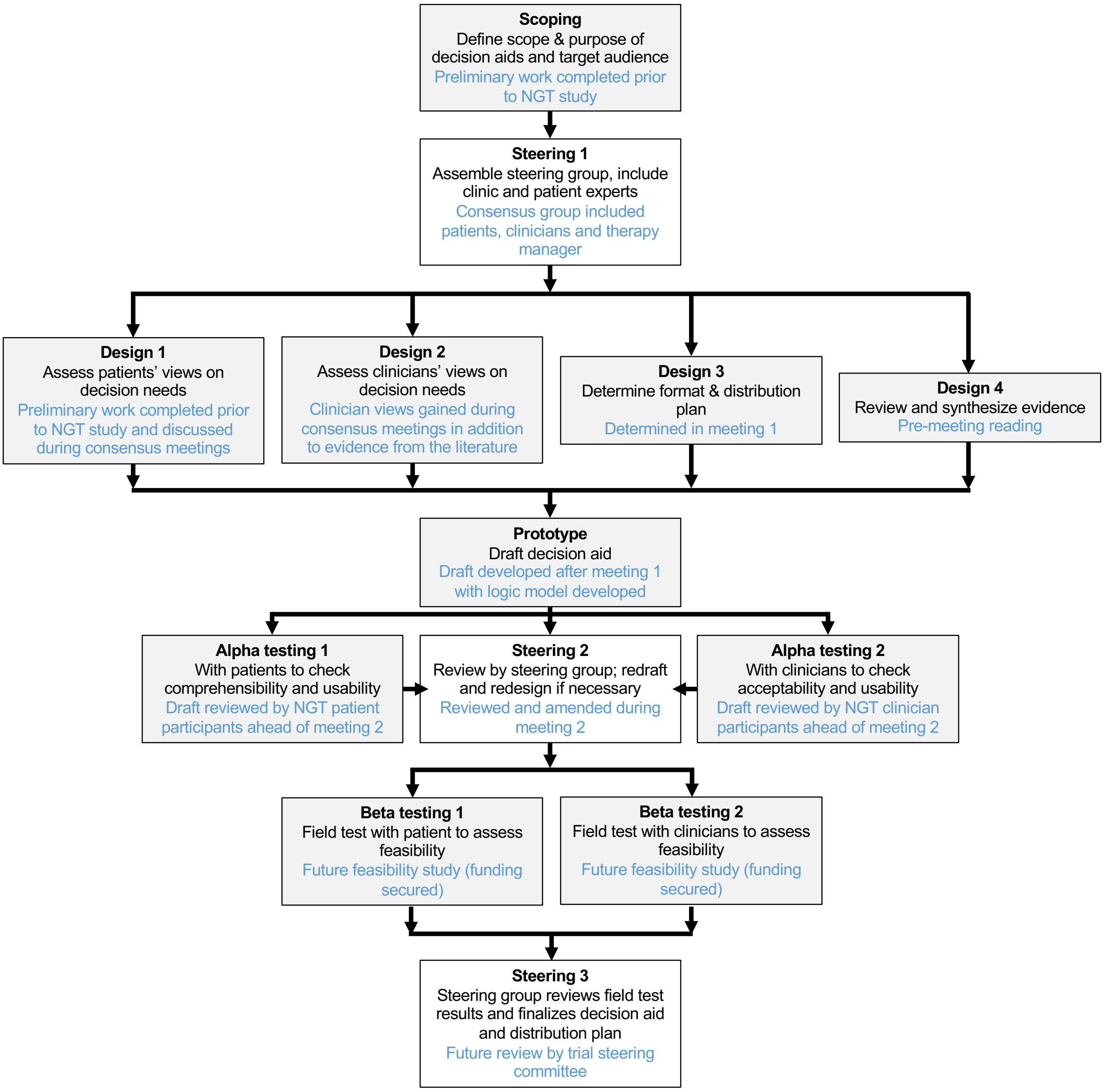

Marrin K, Brain K, Cpsychol P, *et al.* Fast and frugal tools for shared decision-making: how to develop Option Grids. *Eur J Pers Cent Healthc*. 2013;1:240–5. (will do the same as the table for the Elwyn guidance)

**Option Grid Development Process**	**Development of the shared decision-making intervention (Component B, Option Grid)**
Identification of need	Preliminary work completed prior to NGT study
Est. of Editorial Team	NGT participants included patients and key stakeholders
Development of FAQs	Established during meeting 1 and 2
Evidence review	Pre-meeting reading
Population of the Option Grid	Draft composed ahead of meeting 2
Sign-off 1	Signed off by participants after meeting 2
User testing	Future feasibility testing
Review of Option Grid	Review planned following feasibility testing
Sign-off 2	Sign off following feasibility testing
Annual review	To be determined following feasibility and effectiveness testing

National Institute for Health and Care Excellence. Standards framework for shared-decision-making support tools, including patient decision aids | Guidance | NICE. *NICE Corporate Document*. 2021.

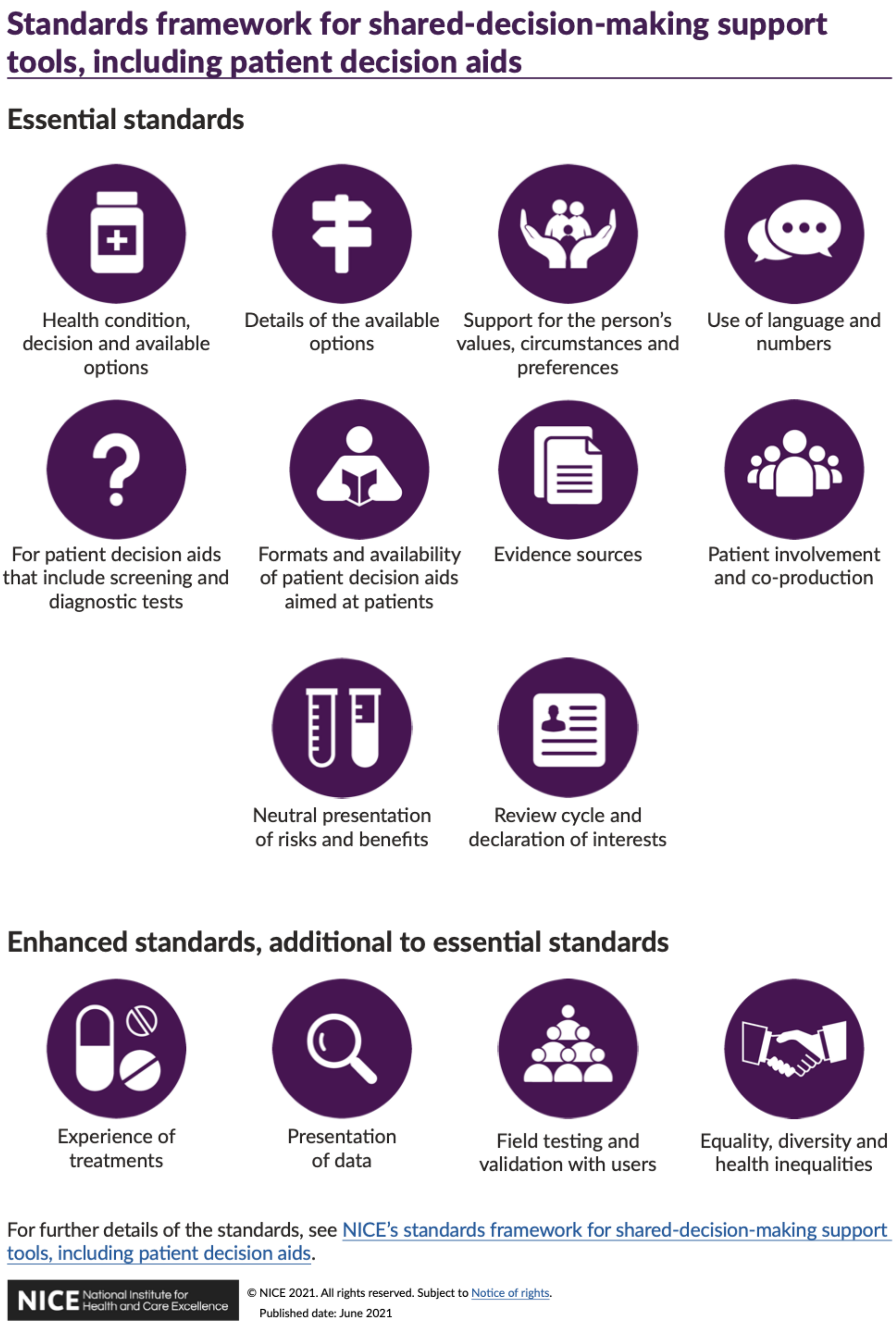

Supplement: online supplemental file 8 [file bmjopen-14-7-s008.pdf]
