# Supplementary material for: Development of an intervention for patients following an anterior cruciate ligament rupture: an online nominal group technique consensus study
Source: BMJ Open. 2024 Jul 18;14(7):e082387. doi: 10.1136/bmjopen-2023-082387 (PMC11261705; doi:10.1136/bmjopen-2023-082387)
Supplement: online supplemental file 9 [file bmjopen-14-7-s009.pdf]

Supplementary File 9

Implementation Research Logic Model

Determinants

Intervention Characteristics

- Evidence & theory-based intervention
- Developed with patients and stakeholders
- Thought to offer an advantage to consultations
- Freely available

Inner Setting

- Clinical consultation
- Implementation readiness

Outer Setting

- ACL pathway → MDT relationships
- NHS → externally driven structures

Characteristics of Individuals

- Patient and HCP knowledge, beliefs, motivation to engage
- Clinician identification i.e.. is it their role
- Health literacy

Process

- Reflective & evaluating
- Quantitative & qualitative feedback
- Refinement

Implementation Strategies

- Education on intervention's use
- Develop with input from & engage with stakeholders
- Understand commitment of those using the intervention
- Adapt & tailor to context
- Ensure adaptable for individual use
- Understand where is fits within the pathway, whose role it is & how it might work
- Clear evaluation strategy to support refinement

Mechanisms

- Recognition of decision
- Trust
- Self efficacy
- Perception of time
- Perception of the patient's health literacy/ability
- Perception to access external support
- Anxiety
- Clinician confidence / motivation / ability
- Balance between patient & healthcare professional relationship
- Understand patient values & preferences
- Built in system support ie. clear fit in the pathway

Outcomes

Implementation

- Feasibility – APEASE
  - Affordability
  - Practicability
  - Effectiveness/cost
  - Acceptability
  - Side-effects/safety
  - Equity
- Reach / uptake
- ENPT

Service

- Effectiveness
- Equity
- Patient centeredness
- Timeliness

Clearer resource ie. right people on the waiting list ?decrease waiting listing

Resource need ie. reduce appointment need from patients, patients chasing clinicians

Clinical/Patient

- Knowledge
- Readiness to make a decision
- Decisional regret
- Involvement congruency / ?perceived involvement in care
- Levels of SDM

Confidence in decision making

Patient centered care

Who does it work for & why?

Implementation strategies mapped to ENPT

Potential

Potential to enact/deliver the intervention

- Engage with stakeholders

Capacity

How does the intervention work in the pathway

- NHS
- Between clinicians – coordination of action(s)

How does the intervention work during consultations between patients and clinicians

Capability

How does the intervention integrate with the NHS system, consultations and pathway

- Strategy = clear about use, when, how
- Adapt & tailor to context

Workability

- Design with key stakeholders
- Paper and online

Contribution

Educate those using it (coherence)

Understand commitment from patients and clinicians (cognitive participation)

Measure engagement (cognitive participation)

Consider work links (collective action)

Appraisal (reflexive monitoring)
