# Supplementary material for: Development of an intervention for patients following an anterior cruciate ligament rupture: an online nominal group technique consensus study
Source: BMJ Open. 2024 Jul 18;14(7):e082387. doi: 10.1136/bmjopen-2023-082387 (PMC11261705; doi:10.1136/bmjopen-2023-082387)
Supplement: Supplementary file 10 [file bmjopen-2023-082387.reviewer_comments.pdf]

PEER REVIEW HISTORY

BMJ Open publishes all reviews undertaken for accepted manuscripts. Reviewers are asked to complete a checklist review form (**http://bmjopen.bmj.com/site/about/resources/checklist.pdf**) and are provided with free text boxes to elaborate on their assessment. These free text comments are reproduced below.

ARTICLE DETAILS

**TITLE (PROVISIONAL)**	Development of an intervention for patients following an anterior cruciate ligament rupture: an online nominal group technique consensus study
**AUTHORS**	Carter, Hayley; Beard, David; Leighton, Paul; Moffatt, Fiona; Smith, Benjamin; Webster, Kate E.; Logan, Phillipa

VERSION 1 – REVIEW

**REVIEWER**	Relph, Nicola<br>Edge Hill University, Department of Applied Health and Social Care
**REVIEW RETURNED**	01-Feb-2024

**GENERAL COMMENTS**	Thanks for the opportunity to review this work. I think this is a very important study that will aid future SDM in ACL treatment. I have listed my minor comments below:<br>1. It would be useful to explain why you chose ENPT to underpin the study in the introduction.<br>2. Explain the NGT briefly in the Method.<br>3. I note that neither of the two patients included in your consensus group had experience of the rehabilitation only pathway (or rehabilitation then surgery). How might this have impacted the tool?<br>4. Should there be a colour code for Figure 3? If not, keep colour consistent.<br>5. Should the information pack include Risks of Rehab after the section on risks of surgery (it is in the decision table but not in the text).

**REVIEWER**	Häger, Charlotte<br>Umeå University, 1Department of Community Medicine and Rehabilitation – Physiotherapy Section
**REVIEW RETURNED**	08-Mar-2024

**GENERAL COMMENTS**	**Development of an intervention for patients following an anterior cruciate ligament rupture: an online nominal group technique consensus study**<br><br>The study addresses important topics in the context of ACL management: shared decision-making and evidence-based recommendations for treatment. The authors attempt to create a shared decision-making intervention to be implemented by clinicians with patients when considering either a conservative or surgical treatment, as well as evidence-based treatment recommendations to be used primarily by clinicians. The study involved eight participants (a musculoskeletal outpatient therapy

	manager, two patients and some clinicians; three physiotherapists, an occupational therapist and a surgeon) and in addition, five non-voting facilitators, all took part in two digital meetings. An online nominal group technique (NGT) was used to develop these tools. This work is targeting timely issues regarding this patient population where there is no consensus for either treatment or delivery.<br><br>**General comments and concerns**<br><br>The study focuses on two important but very wide research questions and tries to answer these by a process of using only two sessions of NGT where no or very little information on the preparation of and the duration of the meetings is provided. It could be questioned whether these sessions including the follow-up work are sufficient for the ambitious purpose of the study, particularly regarding objective number two.<br><br>The participant group in the NGT meetings was very small (thus the generalizability is questioned) and the variety of participants in the group does not seem appropriate to reach each of the two aims of the study. The paper would benefit from a clearer presentation and from explaining the techniques used and the theoretical framework much more including providing relevant references.<br><br>The study could be valuable concerning the aims, but the character is more of a pilot study/preparatory work and should be presented as such. It is not clear what the wider study, already referred to in the Abstract and then again referenced in the Results, exactly is, please explain.<br><br>There are many abbreviations in the text; some are not spelt out at all or used first as abbreviations and spelt out only later in the text (UK, NHS, MSK). Other abbreviations are created with no real need as they are not used in the text or they are used only one or two times (RCT, IPDAS, IRLM, ATOCP, PPIE and more). Using numerous abbreviations might create a more difficult text to read.<br><br>**Specific comments**<br><br>*Abstract*<br><br>The Abstract currently does not provide any background with a clear rationale for the study and why this would be a good method to reach the objectives. A better description of the context of the study might be needed for a better understanding of why this particular study is being done.<br><br>The first objective states that the researchers aim to develop an intervention but it seems like more of an attempt to develop a tool that can be applied in different interventions. There would be a call for clearer definitions and terminology for how the authors consider their efforts/approach. For instance, in the Introduction, on the one hand, the authors exemplify “shared decision-making tools” as a key element of healthcare but at the same time, they seem to regard it as an intervention per se.

	Participants: It's unclear in which role and why the supervisors of the PhD students who are also authors were participating even if non-voting facilitators. What was more precisely the facilitators' role in the study? How much influence did the facilitators have on the study process? Are they co-authors? All of this information does not need to go in the Abstract, but in the Methods, Currently, it only raises more questions than provides answers in the Abstract.<br><br>Setting: wonder whether the current text is appropriate, it is very broad and not explanatory for what it specifically means for an international crowd, maybe a clinical setting with some more information? How does the setting for instance relate to the patient population of the study?<br><br>Maybe Method rather than Intervention as a heading for this study.<br><br>Abbreviations are not spelled out, e.g. (NGT although mentioned under Design, UK could also be spelled out).<br><br>Strength and limitations: The authors only refer to strengths and no limitations. In addition, one mentioned strength was "underpinned by the extended normalisation process theory to ensure implementation factors were considered and embedded within the development process.". This theory is not lifted in the Method section of the Abstract and the reader cannot understand how this theory helps with the development process until having read the paper.<br><br><br>*Introduction*<br><br>The authors refer to the NHS setting in the paper. Although it is a British journal, the readers of the journal are international and might not be familiar with the British system. An example, in line 15, why is the transferability of the described study limited to an NHS setting?<br><br>Some of the sentences could be better phrased (page 6 lines 13-17, 18-22, 29-33).<br><br>The authors could use the same phrasing throughout the paper: page 6 Line 27 – England is used instead of UK, Line 35 - does "theoretically informed" mean evidence-based?<br><br>The target group for the intervention is not presented clearly. In the Abstract (page 4) the objectives state that it is for patients with ACL rupture but when reading the text in the Introduction it seems to be for physiotherapists as well.<br><br>There is only a rationale for the first aim (the SDM intervention). The Introduction does not explain or justify the need for the second

	aim (developing evidence-based treatment recommendations) which would be good even if it may seem intuitive.<br><br>*Methods*<br><br><u>Study design</u>: The study aims to answer two comprehensive questions that might be better answered in two separate and or larger studies. Firstly, the study is based on data from only two meetings with a very small group of participants. Therefore, it could be questioned if these vast research questions could be properly answered (specifically the second objective). Secondly, the recommendation tool (second aim) includes an almost similar SDM tool to that created by the same group of participants at the same meetings (first aim). For this to be convincing based on evidence, not just assumptions of the own teams preferences, this process should be made more transparent.<br><br><u>Study population/ participant group</u>:<br><br>The inclusion criteria state that individuals were considered eligible if they had undergone ACLR or were waiting for one. Since this study aims to build an intervention that supports decision-making for whether or not to undergo surgery, it might be of interest to involve individuals who have chosen not to undergo a reconstruction.<br><br>In addition, this paper addresses two different objectives but it is unclear if the chosen participant group is suitable to answer both. E.g., for the first objective (creation of an SDM tool/intervention), more representatives from the patient population might be needed, as they are the target population of the study.<br><br>The Method section is not described sufficiently or in a logical order for the reader to comprehend easily. There is a lack of information regarding:<br><br>- Nominal group technique method (NGT)- as mentioned above it needs a better description since it's not a widely used method in this research field.<br>- Recruitment of participants is not sufficiently described. Sampling? Strategically?<br>- Information about the data collection; report of the process and full protocol (including detailed and full count of information sent to the participants before each meeting, results of the voting, full report of the discussion of each meeting, etc.). Interview guide? Recording of the process, how? Length of the sessions?<br>- What about the data that didn't reach this cut-off? A clearer description of the pre-elicitation material in general is called for. That is more specifically, what evidence did the participants receive to be able to create evidence-based recommendations? The study refers to many guidelines and models (page 7 lines 10-25). These

	guidelines and models should be better explained (IPDAS, IRLM and even further explanation on ENPT) and added in the supplementary material.<br>- It is also unclear what the non-voter groups' role was; was it just observation or was it active participation in the meetings? How much influence did they have on the voting group?<br>- Additionally, the participant group seems unbalanced with just two patient representatives. This might have created a power imbalance in relation to the experts. Moreover, at the second meeting, only one patient representative participated.<br><br><u>Ethics</u>: what kind of authority or type of committee approved and when. Please clarify.<br><br>*Results*<br><br>The first part of the Results is really Methodology and is recommended to be moved there.<br><br>Some of the results are difficult to understand, perhaps because the methods are not well described.<br><br>Some examples:<br><br>- page 9 line 51- 59, information of what was refined and presented to the participants during the second meeting are presented but do not add to the reader's understanding of the process, since the reader does not know what was discussed and decided during meeting one.<br>- Page 11 "exercise guidelines"- line 9 mentions 9 exercise topics, but it is unclear what are they (figure 2 shows 7 exercise types and Figure 3 shows 7 risks and considerations. Where are the exercise topics explained?).<br>- It is unclear why the specific core components of treatment (i.e., advice and education, exercise guidance delivery method, outcome measures and shared decision-making) have been chosen. Adding a detailed description of the procedure, as well as the reference list to the recommendation tool, is necessary.<br><br>There are 16 topics rated as important in Figure 1, not 15 as stated in the text? Thus 21 in total or?<br><br>A flowchart of the whole study process might facilitate the reader to get a better overview of the study and its results.<br><br>The recommendations are graded as "very important" and "important" but the individuals' autonomy to participate in the choice of treatment is not described. In addition, what does this mean in practice that some of the recommendations are "very important" while others are just "important"? Could the "important" recommendations be disregarded during treatment if there is a lack of time?

	Supplementary material is not numbered and seems to be in a reversed order with regards to what is mentioned under the Results, i.e., the authors are referring to supplementary material 1 and 2 (page 10 line 3-4) as Patient leaflet etc which seems to be wrong as Recommendations are presented as material #1 etc.<br><br>Other comments to this section:<br><br>page 12 figure 3- risks for further injury are addressed here only for those who chose a reconstructive treatment and are mentioned only for the injured leg. The risks for the non-injured leg should be discussed for both treatment options.<br><br>Page 12 “method delivery” please explain what each method delivery means, e.g., what is the difference between one-to-one and face-to-face.<br><br>Page 13 “outcome measures”- different outcome measures are abundant. But it is not sufficient with just the general topic of the outcome measure to be tested (i.e., “knee specific outcome measures” or “clinical assessment”). An evidence-based recommendation should provide more specific evidence-based tools (i.e., what in the clinical examination is crucial: strength, ROM, functional tests, what muscles? ….)<br>Page 13 “shared decision making” – very similar recommendations to the tool developed by the same group during the same meeting. Please expand on this.<br><br>*Discussion*<br><br>What is summarised in the first part of the Discussion is not clearly presented in the Results section, which is a bit confusing.<br><br>The Discussion would preferably include an explanation of the NHS setting and how the developed intervention and recommendations are suitable for this specific setting. This information could also help to apply the results of the study to other settings. Limitations of the study that could be addressed in this section are a small participant group, and the other method limitations in relation to the objectives of the study.<br><br>On page 15 line 35 it is mentioned that the outcomes of this study represent “essential components for inclusion and components to be considered to allow tailoring for individual care.” This is not discussed per se in the Results section. Although the study presents “very important” and “important” recommendations, it does not specify how these recommendations could be used to individualise the care.<br><br>The limitation of power dynamics and imbalance in the group is discussed, but the steps taken to avoid excluding the patients’ voices might not be sufficient. The effect of adding patient facilitator to the non-voter group to support the voting patient

	representatives is questionable. The facilitators were not supposed to argue for their view at the meetings (if the procedure was understood correctly), so their support (especially when digital) might have been very limited. A better solution would maybe have been to include more voting patient representatives.<br><br>Page 16 paragraph 3, line 43, Delete the part starting with Funding is in place…. Irrelevant<br><br>Page 16 line 48-50- The ENPT theory needs to be discussed in more detail, for the reader to understand why this is indeed a strength.<br><br>Page 16 line 50- the term “researcher’s reflexibility” needs to be explained.<br><br><br>*Supplementary materials*<br><br>The researchers used several guidelines that supported their study design and execution. These guidelines, as well as the specific way they were used in this study, should be included.<br><br>Supplementary material should be numbered and presented in the same order as presented in the main text.<br><br><br>*References*<br><br>No references are presented on the “up-to-date literature” the authors provided to the participants (page 8 and line 8).<br><br>Reference for NICE is actually a reference for the IRLM model - page 7 line 13<br><br>Page 15 line 18- reference lacking for the study by Filbay and Grindem, 2019.

VERSION 1 – AUTHOR RESPONSE

Reviewer: 1
Dr. Nicola Relph, Edge Hill University
Comments to the Author:
Thanks for the opportunity to review this work. I think this is a very important study that will aid future SDM in ACL treatment. I have listed my minor comments below:

1. It would be useful to explain why you chose ENPT to underpin the study in the introduction

Thank you for the suggestion to add detail to the choice of ENPT. The final paragraph has been amended which now reads:

“The Extended Normalisation Process Theory (ENPT) was used as the underpinning theory for the study [9]. The use of theory to support intervention development is recommended by the Medical Research Council and National Institute for Health Research framework for developing and evaluating complex interventions, however no single theory has been identified as superior to another [10]. A recent review exploring the normalisation process theory/extended normalisation process theory’s use in interventional research of musculoskeletal/orthopaedic conditions found it to be compatible for use across a variety of conditions and healthcare settings and encouraged it’s use during intervention development processes [11].”

2. Explain the NGT briefly in the Method.

We have added further text to describe the NGT method in more detail. The methods section has also been amended in response to reviewer 2 comments. The description of NGT in the methods now reads:

“NGT uses group meetings comprising of appropriate experts to gain consensus on a topic area [12]. This approach has commonly been utilised in healthcare intervention development [12,13]. Although usually face-to-face, the NGT method was adapted to be conducted online via video teleconference to support collaboration from participants in a spread of geographical locations. This has previously been identified as a pragmatic concern of the face-to-face method [14]. Successful online NGT studies have also been described elsewhere [15]. The online NGT process followed the same structure as described for face-to-face, this is shown in Figure 1.

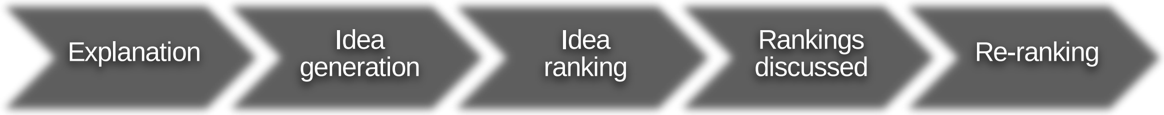

Figure 1 – Nominal group technique process”

3. I note that neither of the two patients included in your consensus group had experience of the rehabilitation only pathway (or rehabilitation then surgery). How might this have impacted the tool?

Thank you for highlighting this as a consideration of participants involved in the study and how this may have impacted the results. We have added this to our limitations section which now reads:

“Further, both patients included in the study had experienced ACLR. This may have influenced patient perspectives on important information to be included in the SDM intervention and treatment recommendations, with a potential preference for surgical intervention. The addition of patient participants with experience of non-surgical management may have offered different perspectives and influenced group discussions and study outputs.”

4. Should there be a colour code for Figure 3? If not, keep colour consistent.

Thank you for suggesting the colour change to ensure consistency, Figure 3 has been amended and is now green to highlight that the risks were voted to be ‘very important’. The figure now appears in the text as below (this is now Figure 5):

Discussion	Balanced presentation	Activity limitations	Empowering patients
**Risks** of engaging in physical activity are discussed with each patient.	A risk and considerations discussion should include a **balanced presentation** of the evidence considering potential **risks and benefits**.	Consider **limiting activities** that provoke knee swelling and instability and cause intolerable amounts of pain.	Ensure risk and consideration discussions **empower patients** to engage in physical activity within the **scope of risk deemed acceptable** to them and their treating clinician.

Exercise guidance	Exercise progression	Risk of further injury
Exercise should be guided by the **appropriate professional** and be within the limits of the individual.	Ensure exercise is **progressive** with **graded exposure** to activities, working towards the patient's goals.	For patient proceeding with surgery, consider the **risks of further injury** and **consequences** to the planned surgical procedure. Consider proceeding with **greater caution**.

5. Should the information pack include Risks of Rehab after the section on risks of surgery (it is in the decision table but not in the text).

Thank you for the suggestion to add the risks of rehabilitation to the intervention. The shared decision-making intervention has now been finalised as we have received ethical approval to explore feasibility, acceptability and implementation factors in a non-randomised feasibility trial with embedded qualitative interviews with patients and clinicians. The intervention was developed as an output of the NGT process with all components included voted as consensus by study participants. We will consider the addition of risks of rehab to the SDM tool patient information leaflet, in line with patient and clinician feedback from the feasibility study.

Reviewer: 2
Prof. Charlotte Häger, Umeå University
Comments to the Author:
Review performed by Sanna Mikko and Yevgenia Grinberg.

General comments and concerns
The study focuses on two important but very wide research questions and tries to answer these by a process of using only two sessions of NGT where no or very little information on the preparation of and the duration of the meetings is provided. It could be questioned whether these sessions including the follow-up work are sufficient for the ambitious purpose of the study, particularly regarding objective number two.

The participant group in the NGT meetings was very small (thus the generalizability is questioned) and the variety of participants in the group does not seem appropriate to reach each of the two aims of the study. The paper would benefit from a clearer presentation and from explaining the techniques used and the theoretical framework much more including providing relevant references.

The study could be valuable concerning the aims, but the character is more of a pilot study/preparatory work and should be presented as such. It is not clear what the wider study, already referred to in the Abstract and then again referenced in the Results, exactly is, please explain.

There are many abbreviations in the text; some are not spelt out at all or used first as abbreviations and spelt out only later in the text (UK, NHS, MSK). Other abbreviations are created with no real need as they are not used in the text or they are used only one or two times (RCT, IPDAS, IRLM, ATOCP, PPIE and more). Using numerous abbreviations might create a more difficult text to read.

We would like to thank you for the careful review of our manuscript and changes which ensure the study process is detailed in more depth. We believe we have responded to the general comments and concerns in the point-by-point responses to the individual comments below. In summary we have:

- Added detail about the NGT process, justifying the sample size and participant inclusion with significant changes to the methods section
- Further explained the theoretical underpinning of the study in the introduction and methods
- Clarified the role of this study in the wider rehabilitation project and how this aligns to the MRC framework for complex intervention development
- Ensured abbreviations are kept to a minimum to support readability

Specific comments

Abstract

The Abstract currently does not provide any background with a clear rationale for the study and why this would be a good method to reach the objectives. A better description of the context of the study might be needed for a better understanding of why this particular study is being done.

We've written the abstract as per the journal's author guidance.

The first objective states that the researchers aim to develop an intervention but it seems like more of an attempt to develop a tool that can be applied in different interventions. There would be a call for clearer definitions and terminology for how the authors consider their efforts/approach. For instance, in the Introduction, on the one hand, the authors exemplify “shared decision-making tools” as a key element of healthcare but at the same time, they seem to regard it as an intervention per se.

Thank you for the comment requesting clarity to the terminology we have used. The shared decision-making intervention comprises of two components which are referred to as tools in the shared decision-making literature. A shared -decision-making tool is an intervention. The intervention was developed following the MRC framework. We have added detail throughout the manuscript to make this clearer by referring to the shared decision-making intervention as an intervention comprising of two tools (the patient information leaflet and option grid).

We are limited with further detail in the abstract due to word count and have set our headings in line with the BMJ Open guidelines. We have made some amendments to the abstract which has hopefully made the context and study processes clearer (responses below).

Participants:
It's unclear in which role and why the supervisors of the PhD students who are also authors were participating even if non-voting facilitators. What was more precisely the facilitators' role in the study? How much influence did the facilitators have on the study process? Are they coauthors?

All of this information does not need to go in the Abstract, but in the Methods, Currently, it

only raises more questions than provides answers in the Abstract.

Thank you for highlighting that the participants section raises multiple questions that aren't appropriate for further detail in the abstract. We have amended the wording in the abstract to avoid confusion and detailed the non-voting participants role further in the methods section. The abstract now reads:

"Consensus meetings comprised of eight voting participants and five non-voting facilitators. Voting participants included: five clinicians, one outpatient therapy manager and two patients with experience of an ACL rupture and reconstructive surgery. Non-voting facilitators supported group discussions and/or observed study procedures. This included a clinical academic expert, two methodology experts and two patient representatives."

The methods section now reads:

"The non-voting participants included: one clinical academic with expertise in orthopaedic and musculoskeletal research including ACL injuries and treatment; two of the lead researcher's PhD supervisors (one with experience of conducting NGT studies and one who is also a clinical academic MSK physiotherapist) and two patient representatives who have been involved in the wider study and it's set up. The clinical academic expert was included to support communication of the most up-to-date literature. The lead researcher's PhD supervisor with experience of NGT methods was present to observe the group, providing oversite to methodological rigour and to support reflexive practices. The patient representatives were involved to support patient participants as they have experience of discussing their views at trial steering committee meetings and PPIE workshops with patients and clinicians."

Setting:

wonder whether the current text is appropriate, it is very broad and not explanatory for what it specifically means for an international crowd, maybe a clinical setting with some more

information? How does the setting for instance relate to the patient population of the study?

The setting has been amended and now reads:

"Online meetings with patients and key stakeholders working and receiving treatment in the National Health Service, United Kingdom."

Maybe Method rather than Intervention as a heading for this study.

The heading has been changed to ‘method’.

Abbreviations are not spelled out, e.g. (NGT although mentioned under Design, UK could also be spelled out).

All abbreviations have been removed from the abstract bar ACL.

Strength and limitations:
The authors only refer to strengths and no limitations. In addition, one

mentioned strength was “underpinned by the extended normalisation process theory to ensure implementation factors were considered and embedded within the development process.”. This theory is not lifted in the Method section of the Abstract and the reader cannot understand how this theory helps with the development process until having read the paper.

Thank you for highlighting that limitations would also be of benefit to be included in this section. We have added a final bullet point which reads:

“Meetings were held online which may have impacted group dynamics, differing the results from that which may have been produced face-to-face. Further, there were more healthcare professionals than patient participants which may have altered the power dynamic of the group.”

We have also added further detail on Extended Normalisation Process Theory’s use in the methods section (see below). Thank you for bringing this to our attention.

Introduction
The authors refer to the NHS setting in the paper. Although it is a British journal, the readers of the journal are international and might not be familiar with the British system. An example, in line 15, why is the transferability of the described study limited to an NHS setting?

Further detail has been added and the text now reads:

“although there is limited transferability of this finding to an NHS setting were expediting surgery is not currently feasible due the backlog in elective orthopaedic procedures following COVID-19)”

Some of the sentences could be better phrased (page 6 lines 13-17, 18-22, 29-33).

These sentences have been amended and now read:

Lines 13-17: “Further, a 2021 randomised controlled trial in the Netherlands concluded that early surgery may be better than delayed surgery (although there is limited transferability of this finding to an NHS setting were expediting surgery is not currently feasible due the backlog in elective orthopaedic procedures following COVID-19) [3]. However, a 2010 randomised controlled trial in Sweden concluded that surgery may not be appropriate for everyone and engaging in rehabilitation as first line treatment may avoid unnecessary surgical intervention [4].”

Lines 18-22: “Understanding which patients are appropriate for surgical or non-surgical treatment is challenging. There is uncertainty over treatment decision making and the sequence of specific treatments and any related temporal factors (i.e. when to undergo specific treatments) continue to be debated.”

Lines 29-33: “Shared decision-making regarding treatment for ACL tears is especially important as there is uncertainty reported in the literature about treatment efficacy.”

The authors could use the same phrasing throughout the paper:

page 6 Line 27 – England is used instead of UK

This sentence refers to the board ‘NHS England’ not the NHS service in the United Kingdom. Each nation of the United Kingdom has a separate board leading the NHS in its respective area (e.g. NHS England, NHS Scotland). To ensure this is clear to the reader we have amended the sentence to read:“ SDM is outlined as a key element of healthcare by both the National Institute of Care and Excellence and NHS England board [5,6].”

Line 35 - does “theoretically informed” mean evidence-based?

Theoretically informed refers to the research being underpinned by relevant theory. This terminology is consistent with that used the MRC guidelines for complex intervention research. This has been added to the introduction which now reads:

“The use of theory to support intervention development is recommended by the Medical Research Council and National Institute for Health Research framework for developing and evaluating complex interventions, however no single theory has been identified as superior to another [10]. A recent review exploring the normalisation process theory/extended normalisation process theory’s use in interventional research of musculoskeletal/orthopaedic conditions found it to be compatible for use across a variety of conditions and healthcare settings and encouraged it’s use during intervention development processes [11]. “

The target group for the intervention is not presented clearly. In the Abstract (page 4) the objectives state that it is for patients with ACL rupture but when reading the text in the Introduction it seems to be for physiotherapists as well.

Thank you for highlighting this, we have amended the objective in the abstract to be clear that the intervention is for use with patients in clinical practice (i.e. between healthcare professionals and patients) which reads:

(1) “To develop an intervention for use in clinical practice to support patients diagnosed with an anterior cruciate ligament (ACL) rupture, with decision-making regarding treatment.

There is only a rationale for the first aim (the SDM intervention). The Introduction does not explain or justify the need for the second aim (developing evidence-based treatment recommendations) which would be good even if it may seem intuitive.

Thank you for the suggestion to add further detail to justify the second aim. We have added a paragraph which reads:

“In addition to uncertainty about treatment decision-making, optimum treatment for patients following an ACL rupture is unknown. Although best practice guidance was jointly published by the British Orthopaedic Association, British Association for Surgery of the Knee and British Orthopaedic Sports Trauma Arthroscopy Association in 2020, detailed recommendations regarding the delivery of advice and education, rehabilitation, mediums of treatment and outcome measure use is not included [8]. Therefore, clinical practice in the United Kingdom varies and optimum treatment is unknown[9].”

Methods

Study design:

The study aims to answer two comprehensive questions that might be better answered in two separate and or larger studies. Firstly, the study is based on data from only two meetings with a very small group of participants. Therefore, it could be questioned if these vast research questions could be properly answered (specifically the second objective). Secondly, the recommendation tool (second aim) includes an almost similar SDM tool to that created by the same group of participants at the same meetings (first aim). For this to be convincing based on evidence, not just assumptions of the own teams preferences, this process should be made more transparent.

We have added further detail in the methods section to explain the study design and sample size, the text now reads:

“Study Design

This study used the nominal group technique (NGT) method to:

1) Develop a SDM intervention for use with patients following diagnosis of an ACL rupture
2) Gain consensus for treatment recommendations covering 5 domains: advice and education, exercise guidance, delivery method, outcome measure use and shared decision-making.

NGT uses group meetings comprising of appropriate experts to gain consensus on a topic area [13]. This approach has commonly been utilised in healthcare intervention development [13,14]. Although usually face-to-face, the NGT method was adapted to be conducted online via video teleconference to support collaboration from participants in a spread of geographical locations. This has previously been identified as a pragmatic concern of the face-to-face method [15] Successful online NGT studies have also been described elsewhere [16]. The online NGT process followed the same structure as described for face-to-face, this is shown in Figure 1.

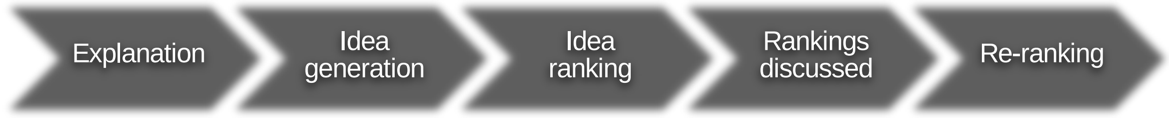

Figure 1 – Nominal group technique process “

Sample Size and Recruitment

Since the development of the nominal group technique method in 1971, there has been debate around the optimal sample size for meetings [17]. More recent literature has proposed 6-12 participants with suggestions that below six will reduce reliability and above 12 will diminish the improvements in reliability [15,18]. We therefore aimed to recruit a sample of 6-12 participants, with a minimum of one participant from each identified professional or patient group. Participants eligible for inclusion included NHS healthcare professionals with a special interest/expertise in treating ACL injuries, therapy managers of an NHS musculoskeletal outpatient department and patients who were awaiting or who had undergone an ACLR.

Participants volunteered to take part in response to study material advertised via professional and special interest networks (Association for Trauma and Orthopaedic Chartered Physiotherapists [ATOCP] and through interested clinicians), social media (Twitter: @POP_ACLR) and an existing patient and public involvement and engagement (PPIE) group. The sampling strategy was to include 6-12 participants with a minimum of one patient, one healthcare professional and one therapy manager. Where more than one potential participant volunteered, their characteristics were considered to ensure variation in group expertise and experience. For example, we aimed to include healthcare professionals from different backgrounds such as medical and allied health professionals.

The consensus group comprised of voting and non-voting participants. Overall, there were 13 participants. The voting participants included: one orthopaedic consultant, three orthopaedic/musculoskeletal physiotherapists, one occupational therapist specialised in vocational rehabilitation in a musculoskeletal outpatient therapy department, one outpatient therapy manager who was also a physiotherapist and had lived experience of an ACLR and two patients with lived experience of an ACL rupture and reconstructive treatment. Non-voters did not partake in voting due to engagement in other research related to this study (this paper reports the intervention development phase of a wider mixed methods study). The non-voting participants included: one clinical academic with expertise in orthopaedic and musculoskeletal research including ACL injuries and treatment; two of the lead researcher's PhD supervisors (one with experience of conducting NGT studies and one who is also a clinical academic musculoskeletal physiotherapist) and two patient representatives who have been involved in the wider study and it's set up. The clinical academic expert was included to support communication of the most up-to-date literature. The lead researcher's PhD supervisor with experience of NGT methods was present to observe the group, providing oversite to methodological rigour and to support reflexive practices. The patient representatives were involved to support patient participants as they have experience of discussing their views at trial steering committee meetings and PPIE workshops with patients and clinicians.

The meetings were chaired by the lead researcher who was a practicing physiotherapist. Three voting participants (surgeon, therapy manager and patient) and one facilitator (PhD supervisor/clinical academic) were unable to make the second meeting due to other commitments. These participants were still included the pre-meeting voting ahead of the second meeting. "

We have added further detail to ensure transparency of our research process. This is under the new heading 'Data Collection and Analysis' which reads:

“Two meetings were held via Microsoft Teams. All participants gave consent to participate in the meetings which were also recorded for study purposes. The first meeting lasted 1hour 51, the second meeting lasted the second meeting lasted 2 hours.

Prior to the first meeting participants were provided with relevant, up-to-date literature in addition to unpublished results from a preceding study which explored patients lived experiences of the ACL surgical treatment pathway pre- and post- surgery using qualitative interviews. All participants received the same information, with a lay document provided to non-clinical participants (available in supplementary file 1 [healthcare professionals] and supplementary file 2 [lay version]). In addition to the information document, pre-meeting voting was conducted to support discussions at the first meeting via Microsoft Forms. Participants were asked to vote on a 5-point Likert scale whether components were ‘very important’, ‘important’, ‘neither important nor not important’, ‘not important’ or ‘not at all important’ for inclusion. Participants voted on five components: (1) advice and education, (2) exercise guidance, (3) delivery method, (4) outcome measures and (5) decision-making. A blank copy of the voting form is available in supplementary file 3. Free text responses were also permitted to stimulate further thought and discussion points to allow room for additional components and topics to be added. This supported the first two stages of the NGT process: explanation and silent generation of ideas.

The purpose of the first meeting was to gain consensus on core components for the recommendations and SDM intervention. A 70% cut-off was used to include components that were considered ‘very important’. Components were deemed ‘important’ if 70% was reached combining scores of very important and important. Components below 30% were excluded and those not reaching the 70% inclusion cut off nor the 30% exclusion were discussed. This threshold was agreed by the study team in line with other consensus recommendations [24,25]. During the meeting Mentimeter was used for anonymous voting and idea sharing. The first meeting consolidated the five components of the recommendations, with no additional components suggested for inclusion. The ‘very important components’ were considered to be essential, whilst the ‘important’ components are recommended to support the personalisation of care. The recommendations are evidence-based and theoretically informed and aim to offer pragmatic guidance to ensure they are achievable, applicable and relevant to the UK NHS setting. The decision-making discussions were split into elements to be included in the recommendations and specific design of the SDM intervention. The need for the recommendations was felt to be important as a direct impact for clinical practice ahead of future feasibility testing of the SDM intervention.

A draft version of the recommendations and SDM intervention was shared with participants ahead of meeting two to provide a basis for group discussion. A second round of voting amongst NGT group participants was conducted prior to the second meeting to support idea sharing and generate discussion. A blank copy is available in supplementary file 4. The second meeting was conducted to agree on specific elements of each component to finalise the recommendations and SDM intervention. The parameters for inclusion were the same as that used for meeting 1. At the end of the second meeting, the overall components and specific elements for the SDM intervention and recommendations were finalised.

The final version of the SDM intervention and treatment recommendations were communicated with all NGT group participants after meeting two (including those who were unable to attend). No concerns

were raised by any participant, so the final versions were considered representative of the discussions and voting across both meetings"

Study population/ participant group:
The inclusion criteria state that individuals were considered eligible if they had undergone ACLR or were waiting for one. Since this study aims to build an intervention that supports decision-making for whether or not to undergo surgery, it might be of interest to involve individuals who have chosen not to undergo a reconstruction.

Thank you for highlighting this, this has been added as a limitation which now reads:

"Further, both patients included in the study had experienced ACLR. This may have influenced patient perspectives on important information to be included in the SDM intervention and treatment recommendations, with a potential preference for surgical intervention. The addition of patient participants with experience of non-surgical management may have offered different perspectives and influenced group discussions and study outputs."

In addition, this paper addresses two different objectives but it is unclear if the chosen participant group is suitable to answer both. E.g., for the first objective (creation of an SDM tool/intervention), more representatives from the patient population might be needed, as they are the target population of the study.

In response to previous comments, further justification regarding participant inclusion and sample size has been added. Further, the study was designed with patient and key stakeholder input which influenced the participant and sample size decisions. This has been added to the patient and public involvement statement section:

"This supported research decision-making regarding participant eligibility criteria, sample size for the study, the research methods and analytical approach."

The Method section is not described sufficiently or in a logical order for the reader to comprehend easily. There is a lack of information regarding:

- Nominal group technique method (NGT)- as mentioned above it needs a better description since it's not a widely used method in this research field.
- Recruitment of participants is not sufficiently described. Sampling? Strategically?
- Information about the data collection; report of the process and full protocol (including detailed and full count of information sent to the participants before each meeting, results of the voting, full report of the discussion of each meeting, etc.). Interview guide? Recording of the process, how? Length of the sessions?
- What about the data that didn't reach this cut-off? A clearer description of the pre-elicitation material in general is called for. That is more specifically, what evidence did the participants receive to be able to create evidence-based recommendations?
- The study refers to many guidelines and models (page 7 lines 10-25). These guidelines and models should be better explained (IPDAS, IRLM and even further explanation on ENPT) and added in the supplementary material.
- It is also unclear what the non-voter groups' role was; was it just observation or was it active participation in the meetings? How much influence did they have on the voting group?
- Additionally, the participant group seems unbalanced with just two patient representatives.

Thank you for suggesting several changes to improve the reporting of our methods sections. We have added further headings to aid readability which include: ethical approval, study design, sample size and recruitment, data collection and analysis, guidelines and supporting theory. We have added detail about the NGT method, further justified and explained our sample size, recruitment and participant involvement, explained the consensus agreement threshold and expanded on the use of the IPDAS, IRLM and ENPT. The methods sections now reads:

Method

Ethical Approval

This research was reviewed and given favourable opinion by the North East - Newcastle & North Tyneside 1 Research Ethics Committee on 27th July 2022 (reference number: 22/NE/0119). The study was registered on clinicaltrials.gov (ID: NCT05529511) on 2nd September 2022.

Study Design

This study used the nominal group technique (NGT) method to:

1) Develop a SDM intervention for use with patients following diagnosis of an ACL rupture
2) Gain consensus for treatment recommendations covering 5 domains: advice and education, exercise guidance, delivery method, outcome measure use and shared decision-making.

NGT uses group meetings comprising of appropriate experts to gain consensus on a topic area [13]. This approach has commonly been utilised in healthcare intervention development [13,14]. Although usually face-to-face, the NGT method was adapted to be conducted online via video teleconference to support collaboration from participants in a spread of geographical locations. This has previously been identified as a pragmatic concern of the face-to-face method [15] Successful online NGT studies have also been described elsewhere [16]. The online NGT process followed the same structure as described for face-to-face, this is shown in Figure 1.

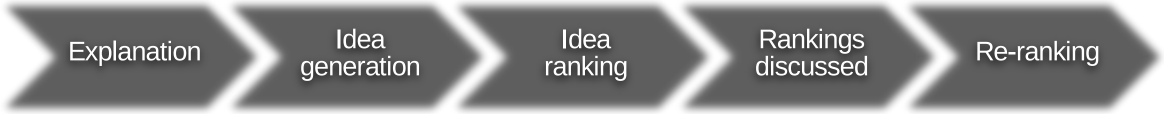

Figure 1 – Nominal group technique process

Sample Size and Recruitment

Since the development of the nominal group technique method in 1971, there has been debate around the optimal sample size for meetings [17]. More recent literature has proposed 6-12 participants with suggestions that below six will reduce reliability and above 12 will diminish the improvements in reliability [15,18]. We therefore aimed to recruit a sample of 6-12 participants, with a minimum of one participant from each identified professional or patient group. Participants eligible for

inclusion included NHS healthcare professionals with a special interest/expertise in treating ACL injuries, therapy managers of an NHS musculoskeletal outpatient department and patients who were awaiting or who had undergone an ACLR.

Participants volunteered to take part in response to study material advertised via professional and special interest networks (Association for Trauma and Orthopaedic Chartered Physiotherapists [ATOCP] and through interested clinicians), social media (Twitter: @POP_ACLR) and an existing patient and public involvement and engagement (PPIE) group. The sampling strategy was to include 6-12 participants with a minimum of one patient, one healthcare professional and one therapy manager. Where more than one potential participant volunteered, their characteristics were considered to ensure variation in group expertise and experience. For example, we aimed to include healthcare professionals from different backgrounds such as medical and allied health professionals.

The consensus group comprised of voting and non-voting participants. Overall, there were 13 participants. The voting participants included: one orthopaedic consultant, three orthopaedic/musculoskeletal physiotherapists, one occupational therapist specialised in vocational rehabilitation in an MSK outpatient therapy department, one outpatient therapy manager who was also a physiotherapist and had lived experience of an ACLR and two patients with lived experience of an ACL rupture and reconstructive treatment. Non-voters did not partake in voting due to engagement in other research related to this study (this paper reports the intervention development phase of a wider mixed methods study). The non-voting participants included: one clinical academic with expertise in orthopaedic and musculoskeletal research including ACL injuries and treatment; two of the lead researcher's PhD supervisors (one with experience of conducting NGT studies and one who is also a clinical academic MSK physiotherapist) and two patient representatives who have been involved in the wider study and it's set up. The clinical academic expert was included to support communication of the most up-to-date literature. The lead researcher's PhD supervisor with experience of NGT methods was present to observe the group, providing oversite to methodological rigour and to support reflexive practices. The patient representatives were involved to support patient participants as they have experience of discussing their views at trial steering committee meetings and PPIE workshops with patients and clinicians.

The meetings were chaired by the lead researcher who was a practicing physiotherapist. Three voting participants (surgeon, therapy manager and patient) and one facilitator (PhD supervisor/clinical academic) were unable to make the second meeting due to other commitments. These participants were still included the pre-meeting voting ahead of the second meeting.

Data Collection and Analysis

Two meetings were held via Microsoft Teams. All participants gave consent to participate in the meetings which were also recorded for study purposes. The first meeting lasted 1 hour 51 minutes, the second meeting lasted 2 hours.

Prior to the first meeting participants were provided with relevant, up-to-date literature in addition to unpublished results from a preceding study which explored patients lived experiences of the ACL surgical treatment pathway pre- and post- surgery using qualitative interviews. All participants

received the same information, with a lay document provided to non-clinical participants (available in supplementary file 1 [healthcare professionals] and supplementary file 2 [lay version]) In addition to the information document, pre-meeting voting was conducted to support discussions at the first meeting via Microsoft Forms. Participants were asked to vote on a 5-point Likert scale whether components were 'very important', 'important', 'neither important nor not important', 'not important' or 'not at all important' for inclusion. Participants voted on five components: (1) advice and education, (2) exercise guidance, (3) delivery method, (4) outcome measures and (5) decision-making. A blank copy of the voting form is available in supplementary file 3. Free text responses were also permitted to stimulate further thought and discussion points to allow room for additional components and topics to be added. This supported the first two stages of the NGT process: explanation and silent generation of ideas.

The purpose of the first meeting was to gain consensus on core components for the recommendations and SDM intervention. A 70% cut-off was used to include components that were considered 'very important'. Components were deemed 'important' if 70% was reached combining scores of very important and important. Components below 30% were excluded and those not reaching the 70% inclusion cut off nor the 30% exclusion were discussed. This threshold was agreed by the study team in line with other consensus recommendations [24,25]. During the meeting Mentimeter was used for anonymous voting and idea sharing. The first meeting consolidated the five components of the recommendations, with no additional components suggested for inclusion. The 'very important components' were considered to be essential, whilst the 'important' components are recommended to support the personalisation of care. The recommendations are evidence-based and theoretically informed and aim to offer pragmatic guidance to ensure they are achievable, applicable and relevant to the UK NHS setting. The decision-making discussions were split into elements to be included in the recommendations and specific design of the SDM intervention. The need for the recommendations was felt to be important as a direct impact for clinical practice ahead of future feasibility testing of the SDM intervention.

A draft version of the recommendations and SDM intervention was shared with participants ahead of meeting two to provide a basis for group discussion. A second round of voting amongst NGT group participants was conducted prior to the second meeting to support idea sharing and generate discussion. A blank copy is available in supplementary file 4. The second meeting was conducted to agree on specific elements of each component to finalise the recommendations and SDM intervention. The parameters for inclusion were the same as that used for meeting 1. At the end of the second meeting, the overall components and specific elements for the SDM intervention and recommendations were finalised.

The final version of the SDM intervention and treatment recommendations were communicated with all NGT group participants after meeting two (including those who were unable to attend). No concerns were raised by any participant, so the final versions were considered representative of the discussions and voting across both meetings.

.

An overview of the study process is shown in Figure 2

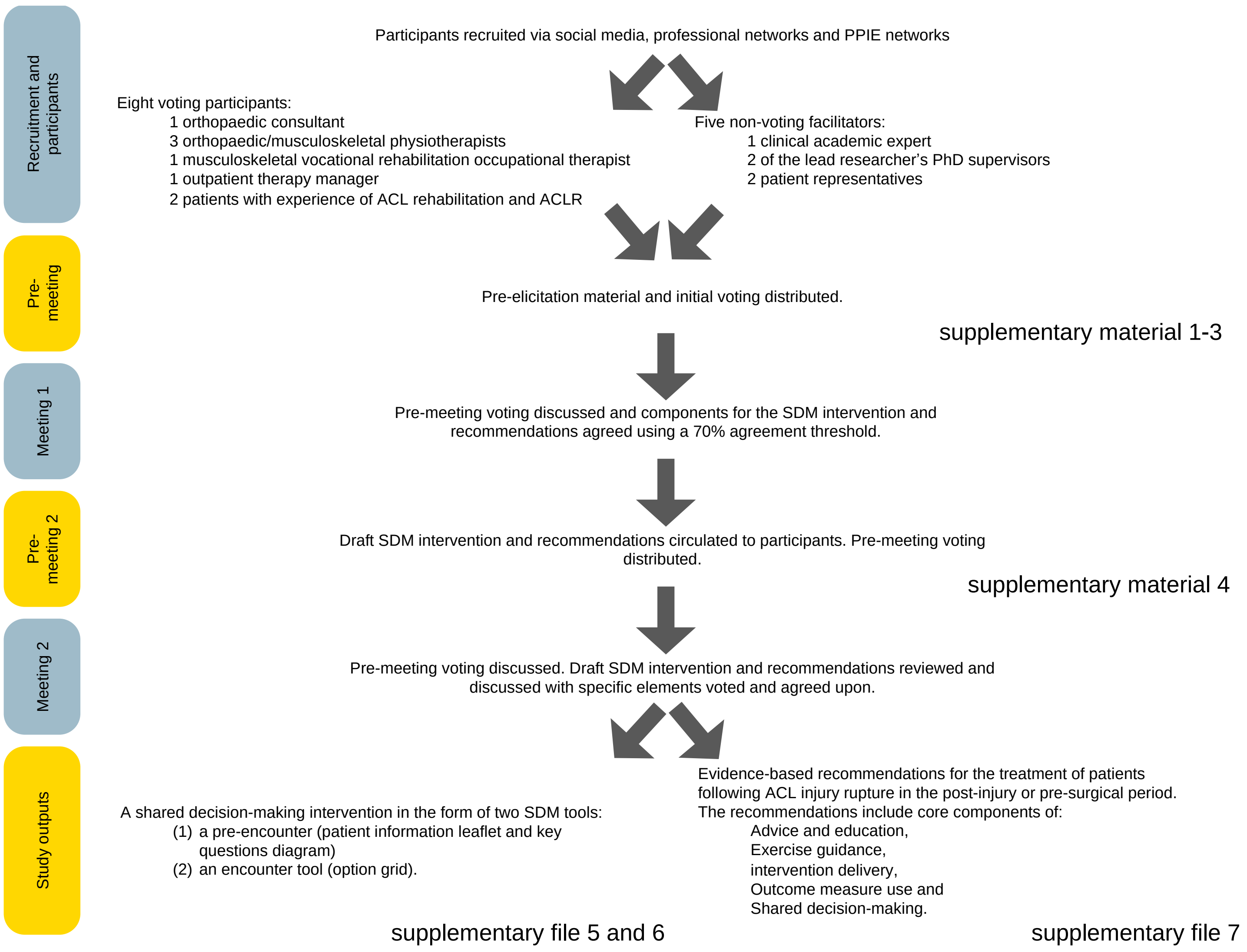
Recruitment and participants
Participants recruited via social media, professional networks and PPIE networks
Eight voting participants:
1 orthopaedic consultant
3 orthopaedic/musculoskeletal physiotherapists
1 musculoskeletal vocational rehabilitation occupational therapist
1 outpatient therapy manager
2 patients with experience of ACL rehabilitation and ACLR
Five non-voting facilitators:
1 clinical academic expert
2 of the lead researcher's PhD supervisors
2 patient representatives
Pre-meeting
Pre-elicitation material and initial voting distributed.
supplementary material 1-3
Meeting 1
Pre-meeting voting discussed and components for the SDM intervention and recommendations agreed using a 70% agreement threshold.
Pre-meeting 2
Draft SDM intervention and recommendations circulated to participants. Pre-meeting voting distributed.
supplementary material 4
Meeting 2
Pre-meeting voting discussed. Draft SDM intervention and recommendations reviewed and discussed with specific elements voted and agreed upon.
Study outputs
A shared decision-making intervention in the form of two SDM tools:
(1) a pre-encounter (patient information leaflet and key questions diagram)
(2) an encounter tool (option grid).
supplementary file 5 and 6
Evidence-based recommendations for the treatment of patients following ACL injury rupture in the post-injury or pre-surgical period.
The recommendations include core components of:
Advice and education,
Exercise guidance,
intervention delivery,
Outcome measure use and
Shared decision-making.
supplementary file 7

Guidelines and Supporting Theory

The International Patient Decision Aid Standards (IPDAS) criteria and option grid development guidelines were used to support the development of the SDM intervention [19–21]. The National Institute for Health and Care Excellence framework for development of decision aids was also used [22]. Visual representation of how these were used is available in supplementary file 8. The Implementation Research Logic Model (IRLM) was used to support visual representation of the SDM intervention considering future implementation and evaluation in a feasibility study [23]. ENPT was used to stimulate discussions during NGT meetings about implementation to embed these considerations in the development process. The four constructs of ENPT were utilised to consider how the intervention would integrate with current NHS systems/pathways (capability), the users' (i.e. clinicians and patients) ability to engage with and deliver the intervention (e.g. considering their resources and skillset) (capacity and potential) and the work and ability of the users to continue engaging with the intervention to allow it to become part of normal practice (contribution). The implementation strategies as part of the IRLM were also mapped to ENPT. The mapping of implementation strategies to ENPT and IRLM is available in supplementary file 9.

Patient and Public Involvement Statement

Patients and key stakeholders (including orthopaedic surgeons, therapy managers and therapists) with experience of having, treating or managing departments treating ACL injuries were involved in the grant funding application, study design and set up. This supported research decision-making regarding participant eligibility criteria, sample size for the study, the research methods and analytical approach."

This might have created a power imbalance in relation to the experts. Moreover, at the

second meeting, only one patient representative participated.

Thank you for highlighting this, we have added this as a limitation (highlighted above)

Ethics:
What kind of authority or type of committee approved and when. Please clarify.

Information regarding ethics has been added to the methods section. The first paragraph now reads:

"This research was reviewed and given favourable opinion by the North East - Newcastle & North Tyneside 1 Research Ethics Committee on 27th July 2022 (reference number: 22/NE/0119). The study was registered on clinicaltrials.gov (ID: NCT05529511) on 2nd September 2022."

Results
The first part of the Results is really Methodology and is recommended to be moved there.

Thank you for suggesting the move of this paragraph. It has been moved to the methods as described above.

Some of the results are difficult to understand, perhaps because the methods are not well described.

Some examples:

- page 9 line 51- 59, information of what was refined and presented to the participants during the second meeting are presented but do not add to the reader's understanding of the process, since the reader does not know what was discussed and decided during meeting one.

In response to previous comments, we have added the voting form as supplementary files 3 and 4.

- Page 11 "exercise guidelines"- line 9 mentions 9 exercise topics, but it is unclear what are they (figure 2 shows 7 exercise types and Figure 3 shows 7 risks and considerations. Where are the exercise topics explained?).

Thank you for highlighting that this could be clearer. We had added information which now reads:

"Exercise Guidance

There were nine exercise topics voted on ahead of and discussed during the first meeting. These included that exercise guidance in the preoperative period should include: at least 1 session with a rehabilitation/exercise therapist, 2-6 sessions with a rehabilitation/exercise therapist, more than 6 sessions with a rehabilitation/exercise therapist, exercise type(s), frequency of exercise completion, exercise programme length, time-period to be completed, delivery method and guidance on exercise/types of activity to avoid."

- It is unclear why the specific core components of treatment (i.e., advice and education, exercise guidance delivery method, outcome measures and shared decision-making) have been chosen. Adding a detailed description of the procedure, as well as the reference list to the recommendation tool, is necessary.

Further detail has been added to the methods section in response to previous comments in addition to further information in the supplementary material.

There are 16 topics rated as important in Figure 1, not 15 as stated in the text? Thus 21 in total or?

There were 15 voted on ahead of the meeting with one topic added during the meeting to total 16. The addition of the voting form in the supplementary material will hopefully make this clearer. We have also added the following text to this sentence:

There were 20 advice and education topics voted on ahead of and discussed during the meeting. Five reached the 70% threshold to be considered as 'very important' for inclusion and the remaining 15 reached the threshold to be considered 'important'. A free text comment suggested the inclusion of spontaneous ACL healing. Following discussion amongst the group and Mentimeter voting, this was considered to be 'important' and so 16 topics were considered to be 'important'.

A flowchart of the whole study process might facilitate the reader to get a better overview of the study and its results.

Thank you for the suggestion to add a study flowchart. This has been included in the methods (described above, figure 2).

The recommendations are graded as “very important” and “important” but the individuals'

autonomy to participate in the choice of treatment is not described. In addition, what does this mean in practice that some of the recommendations are “very important” while others are just “important”? Could the “important” recommendations be disregarded during treatment if there is a lack of time?

Further information to clarify this has been added to the methods which reads:

“The ‘very important components’ were considered to be essential, whilst the ‘important’ components are recommended to support the personalisation of care. The recommendations are evidence-based and theoretically informed and aim to offer pragmatic guidance to ensure they are achievable, applicable and relevant to the UK NHS setting. The decision-making discussions were split into elements to be included in the recommendations and specific design of the SDM intervention. The need for the recommendations was felt to be important as a direct impact for clinical practice ahead of future feasibility testing of the SDM intervention.”

Supplementary material is not numbered and seems to be in a reversed order with regards to what is mentioned under the Results, i.e., the authors are referring to supplementary material 1 and 2 (page 10 line 3-4) as Patient leaflet etc which seems to be wrong as Recommendations are presented as material #1 etc.

Thank you for highlighting this, as further supplementary material has been added this has been reorganised and checked to ensure it aligns with the main text.

Other comments to this section:
page 12 figure 3- risks for further injury are addressed here only for those who chose a

reconstructive treatment and are mentioned only for the injured leg. The risks for the non-injured leg should be discussed for both treatment options.

The risks for the non-injured leg were not discussed amongst the consensus group and therefore have not been added. The intervention is currently undergoing feasibility testing with refinement planned in response to patient and clinician feedback. The risk for the non-injured leg may be included in future versions.

Page 12 “method delivery” please explain what each method delivery means, e.g., what is the difference between one-to-one and face-to-face.

We have added further information to clarify this, which reads:

“It was considered important that the following six mediums are considered by clinicians: (1) booklet, (2) website, (3) face-to-face, (4) one-to-one (via virtual methods or in-person), (5) peer support and (6) combination of face-to-face and digital or printed resources.”

Page 13 “outcome measures”- different outcome measures are abundant. But it is not sufficient with just the general topic of the outcome measure to be tested (i.e., “knee specific outcome measures” or “clinical assessment”). An evidence-based recommendation should provide more specific evidence-

based tools (i.e., what in the clinical examination is crucial: strength, ROM, functional tests, what muscles? ….)

Further information has been added to the text which reads":

"The group agreed upon the recommendation of these outcome measure groups, rather than selecting a specific measure for each category. This was decided to ensure outcome measure selection is most appropriate for individual patients. It further aligns with the ACL literature where several different outcome measures are recommended for use in this population[26]."

Page 13 "shared decision making" – very similar recommendations to the tool developed by the same group during the same meeting. Please expand on this.

Further text has been added which reads:

"Whilst a specific SDM intervention has been developed, it was agreed amongst the group to be beneficial to consider recommendations for SDM principles directly applicable to clinical practice. The need for these recommendations was felt to be important as a direct impact for clinical practice ahead of future feasibility testing of the SDM intervention."

Discussion

What is summarised in the first part of the Discussion is not clearly presented in the Results section, which is a bit confusing.

We have added a summary paragraph at the start of the results to ensure consistency with the first paragraph in the discussion which now reads:

"We present the results for:

1. A shared decision-making intervention comprising of two components: (1) a pre-encounter tool (patient information leaflet and key questions diagram) and (2) an encounter tool (option grid).
2. Evidence-based recommendations for the treatment of patients following ACL injury rupture in the post-injury or pre-surgical period. These recommendations cover core components of advice and education, exercise guidance, intervention delivery, outcome measure use and SDM."

The Discussion would preferably include an explanation of the NHS setting and how the developed intervention and recommendations are suitable for this specific setting. This information could also help to apply the results of the study to other settings.

A paragraph has been added to the discussion outlining the NHS setting, this now reads:

"Both outputs were developed for implementation in the NHS, a public healthcare system unique to the United Kingdom. The transferability to other healthcare systems may therefore be limited. For example, it has previously been reported that patients interact with a median of three different healthcare professionals prior to confirmation of an ACL rupture in the NHS [27]. MRI waiting times are approximately 6-8 weeks and vary between hospitals, with waiting times for surgery currently upwards of 12 months. Whilst in a private healthcare setting, a patient is likely to access an MRI scan

sooner, see fewer healthcare professionals and have the option to proceed with surgical intervention in a shorter timescale."

Limitations of the study that could be addressed in this section are a small participant group, and the other method limitations in relation to the objectives of the study.

Although the sample is small, it is in line with the recommendation in the literature for the NGT method, this has been discussed in the methods. We have acknowledged the disparity in participant type and acknowledged the impact this may have had on the results. The added paragraph reads:

"Further, both patients included in the study had experience ACLR. This may have influenced patient perspectives on important information to be included in the SDM intervention and treatment recommendations, with a potential preference for surgical intervention. The addition of patient participants with experience of non-surgical management may have offered different perspectives and influenced group discussions and study outputs."

On page 15 line 35 it is mentioned that the outcomes of this study represent "essential components for inclusion and components to be considered to allow tailoring for individual care." This is not discussed per se in the Results section. Although the study presents "very important" and "important" recommendations, it does not specify how these recommendations could be used to individualise the care.

This has been added to the methods to clarify the distinction between 'very important' and 'important'. The paragraph in the methods reads:

"The first meeting consolidated the five components of the recommendations, with no additional components suggested for inclusion. The 'very important components' were considered to be essential, whilst the 'important' components are recommended to support the personalisation of care. The recommendations are evidence-based and theoretically informed and aim to offer pragmatic guidance to ensure they are achievable, applicable and relevant to the UK NHS setting."

The limitation of power dynamics and imbalance in the group is discussed, but the steps taken to avoid excluding the patients' voices might not be sufficient. The effect of adding patient facilitator to the non-voter group to support the voting patient representatives is questionable. The facilitators were not supposed to argue for their view at the meetings (if the procedure was understood correctly), so their support (especially when digital) might have been very limited. A better solution would maybe have been to include more voting patient representatives.

This has been added to the limitations as discussed above. The limitations section now reads:

"Further, both patients included in the study had experience ACLR. This may have influenced patient perspectives on important information to be included in the SDM intervention and treatment recommendations, with a potential preference for surgical intervention. The addition of patient participants with experience of non-surgical management may have offered different perspectives and influenced group discussions and study outputs."

Page 16 paragraph 3, line 43, Delete the part starting with Funding is in place…. Irrelevant

This study sits within a larger programme of work developing and then testing the shared decision-making tool. As such, the context in which this study sits is important and we have left this sentence as it stands.

Page 16 line 48-50- The ENPT theory needs to be discussed in more detail, for the reader to

understand why this is indeed a strength.

Further detail regarding the use of ENPT has been added to the methods and supplementary material (supplementary file 9).

Page 16 line 50- the term “researcher’s reflexibility” needs to be explained.

The text has been amended which now reads:

“Author reflexivity (an individual’s reflections of their assumptions, feelings, reactions, judgements) of its use has also previously been recognised to be limited”

Supplementary materials
The researchers used several guidelines that supported their study design and execution. These guidelines, as well as the specific way they were used in this study, should be included.

In response to previous comments, we have added a supplementary file detailing the use of the guidelines referenced. Please see supplementary file 8.

Supplementary material should be numbered and presented in the same order as presented in the main text.

Thank you for reviewing our supplementary material. As we have added further files, this has been amended and checked to ensure consistency with the text.

References
No references are presented on the “up-to-date literature” the authors provided to the participants (page 8 and line 8).

We have included the pre-elicitation material that was distributed to all participants in the supplementary material, which includes relevant references. Please see supplementary file 1 and 2.

Reference for NICE is actually a reference for the IRLM model - page 7 line 13

Page 15 line 18- reference lacking for the study by Filbay and Grindem, 2019.

Thank you for highlighting the error in referencing, this has been amended.

VERSION 2 – REVIEW

**REVIEWER**	Relph, Nicola<br>Edge Hill University, Department of Applied Health and Social Care
**REVIEW RETURNED**	14-May-2024

**GENERAL COMMENTS**	I am happy that my comments have been addressed.
